# Analysis of the transcriptome of the needles and bark of *Pinus radiata* induced by bark stripping and methyl jasmonate

**DOI:** 10.1186/s12864-021-08231-8

**Published:** 2022-01-13

**Authors:** J. S. Nantongo, B. M. Potts, T. Frickey, E. Telfer, H. Dungey, H. Fitzgerald, J. M. O’Reilly-Wapstra

**Affiliations:** 1 School of Natural Sciences, University of Tasmania, Private Bag 5, Hobart, Tasmania 7001 Australia; 2 National Forestry Resources Research Institute, Mukono, Uganda; 3 ARC Training Centre for Forest Value, Hobart, Tasmania Australia; 4 Scion, Rotorua, New Zealand

**Keywords:** Transcriptome, Chemical phenotypes, Bark, Needles, *Pinus radiata*

## Abstract

**Background:**

Plants are attacked by diverse insect and mammalian herbivores and respond with different physical and chemical defences. Transcriptional changes underlie these phenotypic changes. Simulated herbivory has been used to study the transcriptional and other early regulation events of these plant responses. In this study, constitutive and induced transcriptional responses to artificial bark stripping are compared in the needles and the bark of *Pinus radiata* to the responses from application of the plant stressor, methyl jasmonate. The time progression of the responses was assessed over a 4-week period.

**Results:**

Of the 6312 unique transcripts studied, 86.6% were differentially expressed between the needles and the bark prior to treatment. The most abundant constitutive transcripts were related to defence and photosynthesis and their expression did not differ between the needles and the bark. While no differential expression of transcripts were detected in the needles following bark stripping, in the bark this treatment caused an up-regulation and down-regulation of genes associated with primary and secondary metabolism. Methyl jasmonate treatment caused differential expression of transcripts in both the bark and the needles, with individual genes related to primary metabolism more responsive than those associated with secondary metabolism. The up-regulation of genes related to sugar break-down and the repression of genes related with photosynthesis, following both treatments was consistent with the strong down-regulation of sugars that has been observed in the same population. Relative to the control, the treatments caused a differential expression of genes involved in signalling, photosynthesis, carbohydrate and lipid metabolism as well as defence and water stress. However, non-overlapping transcripts were detected between the needles and the bark, between treatments and at different times of assessment. Methyl jasmonate induced more transcriptional responses in the bark than bark stripping, although the peak of expression following both treatments was detected 7 days post treatment application. The effects of bark stripping were localised, and no systemic changes were detected in the needles.

**Conclusion:**

There are constitutive and induced differences in the needle and bark transcriptome of *Pinus radiata*. Some expression responses to bark stripping may differ from other biotic and abiotic stresses, which contributes to the understanding of plant molecular responses to diverse stresses. Whether the gene expression changes are heritable and how they differ between resistant and susceptible families identified in earlier studies needs further investigation.

**Supplementary Information:**

The online version contains supplementary material available at 10.1186/s12864-021-08231-8.

## Introduction

Plants have evolved a variety of constitutive and inducible defences to resist and tolerate herbivory. An assessment of the genetic mechanisms that influence these defences will enhance our understanding of their evolution [1]. Although structural changes in DNA are the major source of genetic variation [2, 3], the phenotypic outcomes of several traits can be linked to gene expression [4–8]. However, the genes and genetic pathways that underlie most phenotypes are still unknown [2]. To date, most gene expression studies have focussed on identifying transcripts (different RNA products a single gene) or genes showing differential expression, or pathways associated with a phenotype (case/control) or condition (treated/untreated). In conifers, for example, transcript abundance has been examined with respect to biotic and abiotic environmental factors such as herbivory [9–11], pathogens [12], artificial wounding [13], drought [14], light intensity [15], seasonal changes [16], chemical stressors like methyl jasmonate [17], as well as associated phenotypic traits such as resistance and chemical composition [9, 10]. Studies in conifer and non-conifer species that have simultaneously compared the expression from different stressors, such as mechanical wounding and methyl jasmonate, indicate both overlapping and non-overlapping gene expression and suggest that molecular mechanisms associated with varying stressors may differ [18–20].

In conifer-herbivory studies, most gene expression studies have focused on understanding induced defence responses, with a premise that these may be more important than constitutive defences as they are metabolically cost effective and expressed only when required [21, 22]. Global transcriptome responses have been studied in both needles and bark, monitoring the expression of a wide range of genes related to the biosynthesis of primary and secondary compounds, and structural components [13, 23–28]. Most of these genes are expressed at basal levels in plants but some are only expressed in the presence of an appropriate stimulus. Some of the genes significantly respond to herbivory cues, by increasing or reducing their expression either locally at the site of the perceived effect or systemically throughout the plant [23, 29, 30]. Studies also show a high overlap in the genes that are differentially expressed when plants are subjected to different biotic and abiotic stresses [31, 32]. However, the genes that show differential expression differ within and between target plant species [10, 26], between plant tissues [23, 33], as well as between biotic agents [34] and applied treatments [35]. Intra-specific differences in the timing of transcript expression have also been observed, where plants may respond to injury within hours or days, with short, or long, lasting effects [17, 23, 25, 33]. Plant responses to different classes of herbivores may differ due to differences in herbivore oral secretions or mode of feeding and the amount of plant tissue damage [34, 36, 37]. While available conifer studies have documented changes in gene expression in response to insect herbivory [13, 32], there are no studies from the perspective of mammalian herbivory, and none that link changes in gene expression to changing chemistry. Mammalian bark herbivory is fundamentally different from insect herbivory in the mode of feeding [22] and possibly the oral secretions. This particularly applies to mammalian bark stripping, which is of increasing concern to managers of conifer forests world-wide, including *Pinus radiata* plantations in Australia [38–40].

*Pinus radiata* is native to California [41], but is now a major plantation species in Australia (ABARES 2018) where it is subject to bark stripping, mainly by native marsupials (wallabies and kangaroos) [42]. The bark is stripped from the base of the trees during the early stages of growth [43–45], reducing tree growth rate, distorting stems and, in extreme cases, causing death [38, 42]. The levels of bark stripping within plantations may be highly variable and progeny trials have shown a genetic, physical and chemical basis to this variation [42, 46, 47]. Further, chemical profiling in *P. radiata* shows that needles and bark respond differently to bark stripping and other forms of real and simulated herbivory, mostly by increasing levels of secondary compounds, especially terpenes and phenolics [48, 49], and reducing levels of sugars and fatty acids [46, 50]. This suggests changes in the expression of underlying genes that subsequently transforms the chemical phenotype. Indeed, the differences in timing of the induced changes in terpenes, phenolics and sugars [50–52] suggest corresponding differences in the expression of the underlying genes. However, while transcriptomic changes have been studied in *P. radiata* associated with ontogeny, wood formation [53–55] and fungal infections [56], those underlying the induced chemical changes to bark stripping have not been characterised.

The present study aims to quantify and compare the transcriptome changes that occur in response to artificial bark stripping of *P. radiata* and whole plant stress induced by application of the chemical stressor, methyl jasmonate. The longer-term goal is to identify genes that specifically mediate the previously shown induced chemical responses to bark stripping in *P. radiata*, which may help develop strategies to reduce bark stripping. The specific aims of the study are to: 1) characterise and compare the constitutive transcriptome of *P. radiata* needles and bark; 2) identify genes which are differentially expressed following artificial bark stripping (aimed at mimicking mammalian bark stripping); and 3) identify genes which are differentially expressed following whole plant application of methyl jasmonate and compare these induced responses with those of bark stripping. The results are discussed in view of the holistic chemistry that has been characterised on the same individuals with the same treatments [50].

## Materials and methods

### Experimental design

In 2015, 6-month-old seedlings from 18 full-sib families (each with 4 seedlings; total number of seedlings = 72) of *P. radiata* (D. Don) originating from the Radiata Pine Breeding Company deployment population, were obtained from a commercial nursery. Seedlings were transferred into 145 mm × 220 mm pots containing 4 L of basic potting mix (composted pine bark 80% by volume, coarse sand 20%, lime 3 kg/m^3^ and dolomite 3 kg/m^3^) and raised outdoors in a common fenced area (to protect against animal damage) at the University of Tasmania, Hobart. At 2 years of age, plants were moved to a shade house and an experimental design established by randomly allocating the 18 families to three treatment groups (methyl jasmonate [MJ], artificial bark stripping-strip [strip] and control), each with 6 families. The three treatment groups were arranged in a randomized block design of 3 blocks, each block comprised a treatment plot of two families, with the treatment plots separated within each block to minimise any interference among treatments. Each family was represented by four plants arranged linearly, and randomly allocated to four sampling times (T0-T21). T0 represents the time immediately before treatment applications. T7, T14 and T21 represent respective sampling times at 7, 14 and 21 days after treatment (MJ and strip) application. All T0 seedlings (*n* = 18), irrespective of group allocation, were not treated and were used to compare the constitutive transcriptome of the needles and bark (i.e. plant parts). Additionally, all seedlings allocated to the control were not treated throughout the experimental period. One seedling from each family in the control and treated groups was destructively sampled at each sampling time to estimate differential expression (*n* = 18; Table 1). For each plant part, comparisons were made between the control (*n* = 6) and methyl jasmonate (MJ, *n* = 6) and between the control (*n* = 6) and bark stripping (strip, *n* = 6) treatments at each sampling time (T7, T14, T21) (Table 1). Methyl jasmonate (MJ) was applied in a 25 mM solution by spraying the whole plant with a fine mist from a hand sprayer until ‘just before run-off’. The treated seedlings were sprayed in a well-ventilated area away from untreated seedlings to avoid cross contamination [57]. For bark stripping (strip), 18 plants were artificially stripped by removing a 30 cm vertical strip of bark, beginning 2 cm from the ground and covering 50% of the stem circumference, which is the average upper threshold of browsing observed in natural field conditions.

**Table 1:** The treatments, sample size and pairwise comparisons that were made for each time and for the two treatments - bark stripping (strip) and methyl jasmonate (MJ). The seedlings of each family were grown in a line-plot and one was chosen at random for destructive harvesting at each time (T7 to T21). At T0, the sampled seedlings were destructively harvested just before treatment applications. At 7 (T7), 14 (T14) and 21 (T21) days after treatment, one seedling from each family (total number of seedlings per sampling time = 18, equivalent to the number of families and *n* = 6 are seedlings selected from each treatment) was destructively harvested

	Control	MJ	Strip	Total # seedlings sampled at each time	
	# seedlings	# seedlings	# seedlings		
T0	6	6	6	18	Sampled before application of treatments, for constitutive transcriptome analysis
T7	6	6	6	18	sampled 7 days after treatment application
T14	6	6	6	18	sampled 14 days after treatment application
T21	6	6	6	18	sampled 21 days after treatment application
Total # seedlings for each treatment	24	24	24	72	

Up to 20 young needles were randomly collected per seedling from different parts of the crown. The bark was sampled from different points of the stem, above and besides the area where the bark stripping treatment was applied, carefully avoiding the wood, following Nantongo et al. [50]. Individual samples were kept separate providing 144 samples for sequencing (2 plant parts × 72 seedlings). The needles and bark samples were snap frozen in liquid nitrogen and were stored at − 80 °C until RNA extraction. The 6 families sampled from each treatment at each time point were treated as biological replicates. No technical replicates were included. This sampling occurred at the same time when the tissue for the chemistry assays reported in Nantongo et al. [50] was sampled.

### RNA extraction and sequencing

RNA from all the 144 bark and needle samples was extracted using the Spectrum™ Plant Total RNA kit (Sigma Aldrich, St. Louis, Missouri, USA, lot # SLBW2113). The RNA extraction was random with respect to part, sampling time, treatment, family and shade house replicate. The quality and quantity of the RNA extracts were assessed with an Agilent 5200 Fragment Analyzer (Palo Alto, California, USA). One sample had poor quality RNA and was excluded from further processing. Using the high-quality RNA samples, 143 separate libraries were prepared with a 6-bp nucleotide bar-coding tag for each library. To construct the library, approximately 1 μg of total RNA was used following the MGIEasy RNA Directional Library Prep Kit (MGI, China). Paired-end sequencing was performed using the Beijing Genomics Institute, (BGI, China) MGISEQ-2000 sequencer according to the manufacturer’s instructions, yielding 100-bp paired-end reads and a total of 20 m reads per sample. Tagged cDNA libraries were sequenced in separate lanes. The library for each lane was selected at random. The quality of RNAseq sequences was assessed using FastQC version 0.11.8 [58]. Quality trimming and filtering of data was performed using Trimmomatic v 0.39 [59]. On average, 99.9% of the sequences were retained at phred33 [60].

A de novo assembly of the pooled transcriptome was attempted using TRINITY v2.9.0 using default parameters [61], however due to the excessive computation requirements, it could not be completed with the available resources in the required timeframe. Accordingly, the filtered reads were aligned to the *P. radiata* reference transcriptome that is harboured at Scion (the New Zealand Forest Research Institute trading as Scion, Rotorua New Zealand) [54] with SALMON v0.14.1 using default parameters [62]. This reference transcriptome (www.ncbi.nlm.nih.gov/bioproject/482145) was assembled from a range of *P. radiata* genotypes and tissue types that were collected at different developmental and temporal stages. Most of the samples were from healthy seedlings under normal growth conditions but also included some pathogen infected seedlings [54]. The reference transcriptome has a total of 279,510 unique transcripts.

### Differential transcripts expression analysis

Statistical analysis of differential expression was performed using the edgeR v3.24.3 package in R (v3.6.0) [63] using default parameters [64], except for the cut-off false discovery rate (FDR) in treated samples that was modified as described below. EdgeR uses the Poisson distribution model to examine differential expression of replicated count data, which makes it simpler than methods that use other statistical distributions [65]. Transcripts were first filtered retaining only those with a minimum expression change of 2 fold and with a minimum of 100 counts per million of a single transcript in at least two part x treatment x time groups. To adjust for library sizes and skewed expression of transcripts, the estimated abundance values were normalized using the trimmed mean of M-values normalization method included in edgeR. To detect differential transcript expression between the needles and the bark, the samples taken at T0 were used as these comprised a single plant from each of the 18 families (as treatments were not applied at this stage) and an FDR value of 0.05 was used. However, to establish transcript expression after treatment, instead of using an FDR of 0.05, a more conservative sample-specific approach was used [66], where transcript expression was initially compared between the samples collected from the control plants (*n* = 6), MJ-allocated (*n* = 6) or strip-allocated (*n* = 6) groups at T0 (before treatment) to check the inherent (potentially random) differences between sample groups. The *p*-values at which no differential expression was detected between these groups was set as the FDR for downstream pairwise comparisons. Accordingly, the *p*-value for detecting differentially expressed transcripts (DET) in the treated needles following both MJ and bark stripping was set at 1.0 × 10^− 11^. A *p*-value of 1.0 × 10^− 18^ was set to detect DET in MJ treated bark and 1.0 × 10^− 10^ to detect DET in the bark stripped samples. Twelve pairwise comparisons were performed. An upset diagram was generated using the UpSetR function in R to summarise the transcripts that were identified as significantly differentially expressed across different comparisons.

Principal component and unsupervised cluster analyses were performed to detect the dominant, relative expression patterns across the needles, bark and treatments. Following Ralph et al. [13], a subset of 500 transcripts with the highest variability and highest expression across the 143 libraries were selected in edgeR for this analysis. Principal components analysis (PCA), using FactoMinerR version 1.41 [67] was based on the correlation matrix among all identified transcripts. Clustering and heat maps were generated using the heatmap.2 function from the gplots package in R, with a matrix of Euclidean distances from the log2 counts of normalised transcripts.

### Sequence similarity search

For sequence similarity search and functional analysis of differentially expressed transcripts (DETs) the transcripts were blasted against the nucleotide BLAST database using BLASTn (https://blast.ncbi.nlm.nih.gov/Blast.cgi). BLAST analysis revealed that *P. radiata* transcripts were most similar to those predicted from genome sequences of *P. taeda* (BLASTn with e- value < 0.0001). Other species, mostly *P. sylvestris*, *P. monticola*, *Picea stichensis and Pseudotsuga menziesii*, showed high similarity with the *P. radiata* transcripts. Annotations of selected transcripts were done by comparing *P. radiata* transcripts to the sequences in the SwissProt database of annotated genes [68] using cut-off values ≤ 1. To gain clear patterns of the responses, only transcripts associated with genes of known function were included. However, there were many uncharacterised transcripts and proteins of unknown functions.

### GO classification

Gene ontology (GO) classification was undertaken to understand the biological process, cellular component and molecular function categories represented in the genes exhibiting differential expression. These assignments were done for selected transcripts identified above using protein analysis through evolutionary relationships (PANTHER) version 14.1 [69]. This was first undertaken using transcripts that were differentially up-regulated in the needles over the bark and vice versa, with the aim of understanding the constitutive differences of the GO processes between the transcriptome of the needles and the bark. Secondly, the GO classification was performed on selected T1 transcripts to understand the differences in the up-regulated and down-regulated transcripts after treatment, as well as differences in the induced transcriptome of the strip and MJ treated samples. Due to the limited annotation resources available for conifers, gene family annotations were obtained using genomes of 10 species: *Arabidopsis thaliana*, *Citrus sinensis*, *Cucumis sativus*, *Oryza sativa, Populus trichocarpa*, *Prunus persica, Saccharomyces cerevisiae*, *Theobroma cacao, Vitis vinifera* and *Zea mays*. GO term classification was done for the top differentially expressed transcripts in the different conditions (time × treatment × part).

## Results

### The *Pinus radiata* reference transcriptome and read mapping

RNA-seq of *P. radiata* generated a total of 2860 million 100-bp PE reads with a minimum of 20 million reads from each of the 143 samples. 87.6% of the reference transcriptome was represented among the study transcripts. However, after the filtration criteria described above, only 6312 unique transcripts (2.6% of the reference transcriptome) were retained as the expression of the other transcripts was too low. The analysis was constrained to individual transcripts, which may not be unigenes.

### Differential expression of the transcriptome

**Fig. 1:**
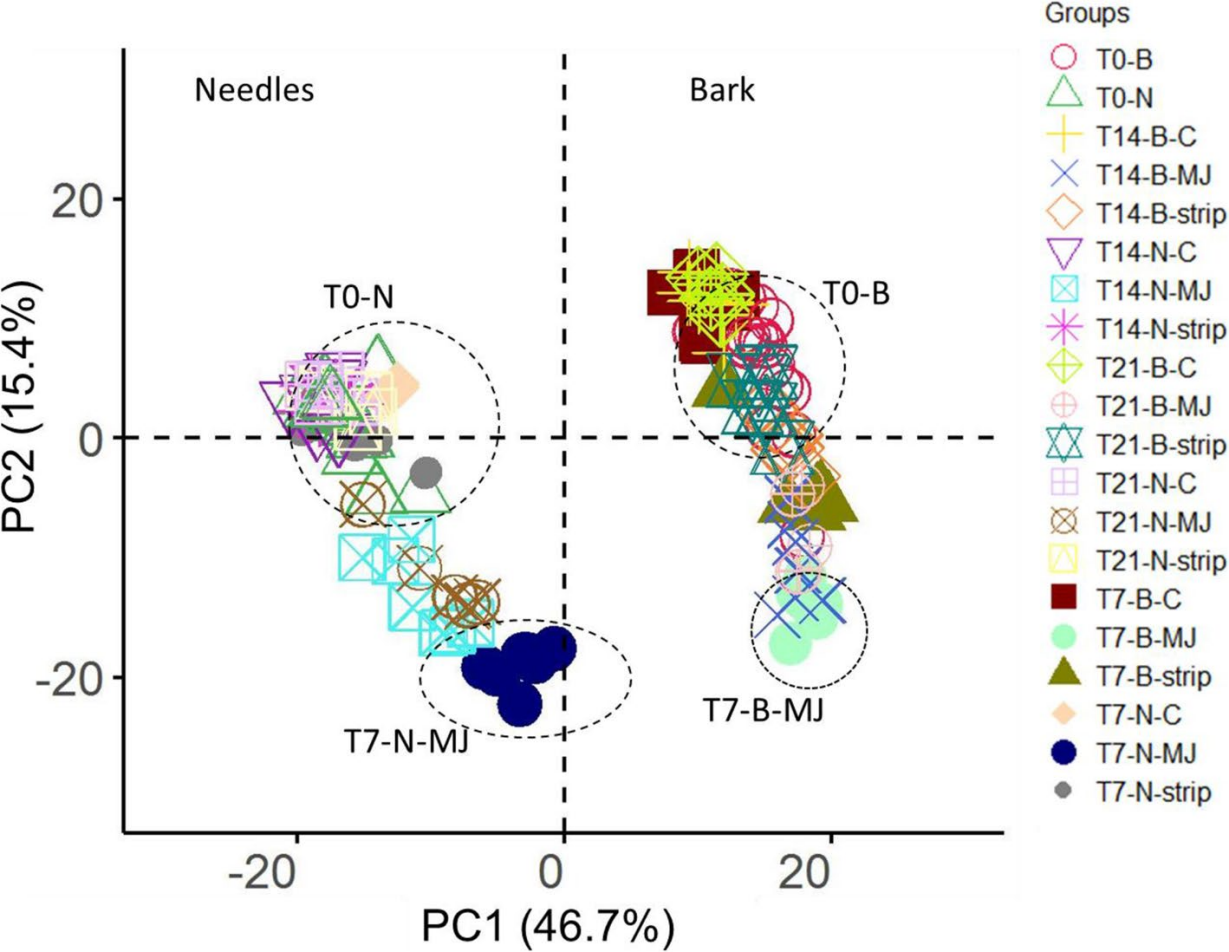
PC1 versus PC2, each explaining 46.7 and 15.4% respectively of the total variation among the 143 samples sequenced based on the 500 transcripts with the highest variability among the samples and highest expression. The samples include the untreated bark (B) and needle (N) controls (circled T0-N and T0-B) and samples from plants treated with bark stripping (strip) as well as methyl jasmonate (MJ) (circled T7-N-MJ and T7-B-MJ)

**Fig. 2:**
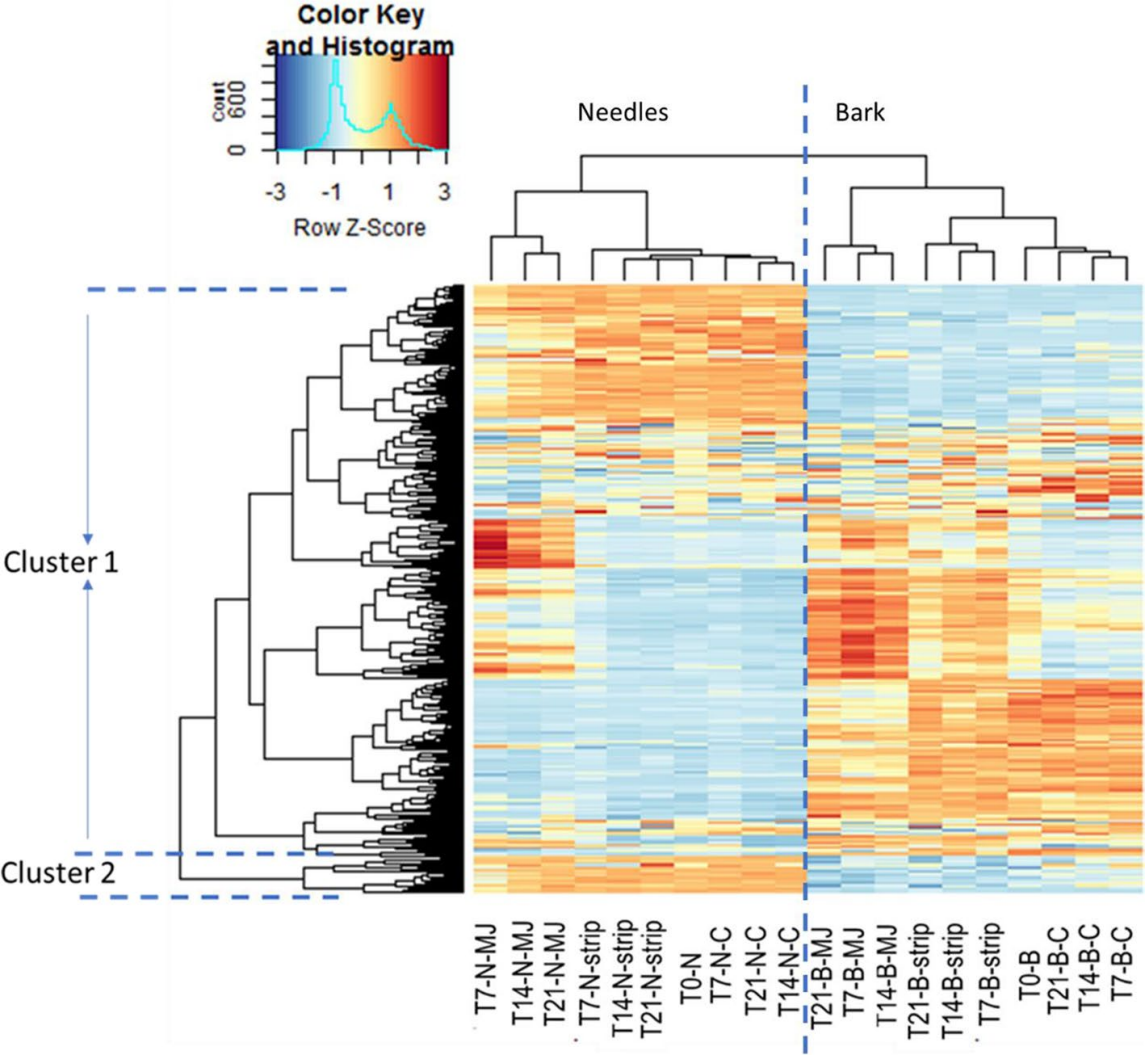
Hierarchical cluster analysis of the top 500 most variable transcripts selected by edgeR in the needles (N) and bark (B) treated with methyl jasmonate (MJ) and artificial bark stripping (strip) and control (C), 7 (T7), 14 (T14) and 21 (T21) days after treatment application. Transcripts (rows) and time/part/treatment categories (columns) were clustered using Euclidean distance. The Z-score is calculated by subtracting the trimmed mean of the M-value of the individual from the grand mean of all the individuals and then dividing by the standard deviation. Trimmed Means of M values are estimated in edgeR by where highly expressed genes and those that have a large variation of expression are excluded, whereupon a weighted average of the subset of genes is used to calculate a normalization factor. Colouration; yellow = mean expression, blue = expression below the mean and red = expression above the mean. The categories on the x-axis were re-arranged based on similarity

The overall relationships between the transcriptome from the different samples were visualised using a principal component analysis (PCA) plot (Fig. 1) and the unsupervised hierarchical clustering (Fig. 2) of the top 500 variable transcripts in the transcriptome. Both figures show that the major differences in expression were due to plant parts (differences along the x-axis of Fig. 1 and the top x-axis of Fig. 2). Within plant parts, we noted genes that were:

(i) up-regulated in the needles relative to the bark and generally non- responsive to treatment;
(ii) up-regulated in the bark relative to the needles and generally non-responsive to treatment;
(iii) up-regulated in either the needles or the bark and responsive to treatment; and
(iv) not differentially expressed between the needles and the bark but responded to treatment by up- or down-regulation.

### Differences in the constitutive needle and bark transcriptome

Of all 6312 transcripts considered for analysis, 5 transcripts were detected only in the needles and 13 transcripts were detected only in the bark. Most of these part-specific transcripts were uncharacterised (Table 2). Gene level annotation of the top 10 transcripts expressed in each plant part are listed in Table 3 (superscript refers to ID number in Table 3). The type 2 light-harvesting chlorophyll a/b-binding polypeptide^[1]^ that is possibly involved in photosynthesis, was the most expressed gene in both the needles and the bark and was represented by different copies of transcripts (isoforms). The needles had other photosynthesis-related genes expressed such as ribulose bisphosphate carboxylase/oxygenase (RuBisCO)^[12]^ and PSI-D1 precursor^[17]^ possibly due to its major role in photosynthesis. Genes related to secondary metabolism were also detected among these top 10 genes, suggesting that constitutive defence is important in *P. radiata*. These included dehydrin^[2]^, metallothionein^[3]^, chalcone synthase^[4]^, defensin^[5]^ and pathogenesis-related proteins^[8]^ and were represented by more transcripts in the bark than in the needles but their relative expression was not statistically significantly different between the needles and the bark.

**Table 2:** Transcripts that were unique to each *Pinus radiata* plant part in the constitutive transcriptome as assessed at T0 (sampled before treatment). The Scion transcript code, predicted gene name and predicted functions of the known genes are indicated

Scion transcript code	Gene name	Gene function
*Transcripts expressed in the needles but not in the bark at T0*		
NZPradTrx008090_C01	Unknown	
NZPradTrx102814_C01	Hypothetical protein 0_2136_01	
NZPradTrx114705_C04	PREDICTED: uncharacterized LOC101213828	
NZPradTrx119356_C01	Repetitive proline-rich cell wall protein 2 precursor, putative	Key determinant of many cell wall proteins https://www.uniprot.org/uniprot/Q40375
NZPradTrx138443_C01	Unknown	
*Transcripts expressed in the bark but not in the needles at T0*		
NZPradTrx105287_C05	Chloroplast ELIP early light-induced protein	Prevents photooxidative stress (Hutin et al. 2003)
NZPradTrx068786_C02	Unknown	
NZPradTrx110900_C02	Unknown	
NZPradTrx158724_C01	Unknown	
NZPradTrx111161_C02	Embryo-abundant protein	May act as a cytoplasm protectant during desiccation. https://www.uniprot.org/uniprot/P46520
NZPradTrx032755_C01	Unknown	
NZPradTrx054373_C01	Unknown	
NZPradTrx151188_C01	Unknown	
NZPradTrx007008_C01	Unknown	
NZPradTrx069030_C01	Unknown	
NZPradTrx081218_C01	Unknown	
NZPradTrx154223_C01	PREDICTED: tetrahydrocannabinolic acid synthase-like	Catalyzes the oxidative cyclization of the monoterpene moiety in cannabigerolic acid https://www.uniprot.org/uniprot/Q8GTB6
NZPradTrx189870_C01	Uninformative

**Table 3:** Top most expressed transcripts (identified by the percentage number of transcripts represented) in the constitutive transcriptome of the bark and the needles as assessed at T0 (sampled before treatment), indicating their identification number, Scion transcript code, gene name and predicted function. Some transcripts were represented by different copies of the transcripts (isoforms—- represented by different transcript codes in each row) and the percentages of transcripts represented by each isoform are indicated. Each isoform has a superscript linking it to its corresponding percentage number of transcripts identified. Ba = first isoform identified in the bark for the gene, Na = first isoform one identified in the needles etc. The transcripts were not significantly differentially expressed between the bark and the needles. Some transcripts were selected in both plant parts

ID number	Scion transcript code (or isoforms)	Gene name	Predicted gene function	Percentages of transcripts (out of 6312)	
				Bark	Needles
1	NZPradTrx107583_C02 ^Ba, Na^	Light-harvesting chlorophyll a/b-binding polypeptide (Lhcb2) mRNA	Absorb sunlight and transfer the excitation energy to the core complexes of PSII in order to drive photosynthetic electron transport (Liu et al. 2013) [70, 71]	1.46 ^Ba^,0.28 ^Bb^, 0.25 ^Bc^	1.99^Na^, 0.95 ^Nb^, 1.07 ^Nc^, 0.51 ^Nd^, 0.51 ^Ne^, 0.33 ^Nf^,
	NZPradTrx050124_C01 ^Bb, Nb^				
	NZPradTrx118940_C01 ^Bc, Nc^				
	NZPradTrx107583_C01 ^Nd^				
	NZPradTrx107583_C03 ^Ne^				
	NZPradTrx050061_C01 ^Nf^				
2	NZPradTrx100458_C02 ^Ba^	Dehydrin 7	Involved in dehydration stress (Stival Sena et al. 2018) [72]	1.38 ^Ba^, 0.60 ^Bb^	
	NZPradTrx100458_C03 ^Bb^				
3	NZPradTrx112612_C02 ^Ba, Na^	Metallothionein 3	Play important roles in metal homeostasis and protection against heavy metal toxicity (Nevrtalova et al. 2014) [73]	0.82 ^Ba^,0.29 ^Bb^	0.58 ^Nc^,1.75^Na^, 0.66 ^Nb^
	NZPradTrx085990_C02 ^Bb^				
	NZPradTrx094970_C01 ^Nb^				
	NZPradTrx094970_C02 ^Nc^				
4	NZPradTrx052720_C01 ^Ba^	Chalcone synthase	Plays crucial roles in phenolic biosynthesis (Dixon and Paiva 1995) [74]	0.70 ^Ba^, 0.37^Bb^, 0.35 ^Bc^,0.27 ^Bd^, 0.26 ^Be^	0.30 ^Na^
	NZPradTrx115271_C03 ^Bb^				
	NZPradTrx078806_C01 ^Bc, Na^				
	NZPradTrx115271_C02 ^Bd^				
	NZPradTrx115271_C05 ^Be^				
5	NZPradTrx050994_C02 ^Ba^	Defensin	Inhibit the growth of a broad range of pathogens, including bacteria, fungi and viruses (Ermakova et al. 2016; Picart et al. 2012) [75, 76].	0.61 ^Ba^, 0.53 ^Bb^	
	NZPradTrx050994_C01 ^Bb^				
6	NZPradTrx076819_C01	TCTP-like protein	Implicated in important cellular processes, such as cell growth, cell cycle progression, malignant transformation and in the protection of cells against various stress conditions and apoptosis (Bommer and Thiele 2004)	0.42	
7	NZPradTrx062252_C01 ^Ba^, NZPradTrx107621_C01 ^Bb^	Nonspecific lipid transfer protein	Play important roles in resistance to biotic and abiotic stress. Have the ability to bind or transfer various types of hydrophobic molecules in vitro, such as fatty acids, fatty acyl-CoA, phospholipids, glycolipids and cutin monomers (Liu et al. 2015a)	0.27 ^Ba,^ 0.26 ^Bb^	
8	NZPradTrx116410_C12	Pathogenesis-related protein 10	Show biological activities related to disease resistance (Liu and Ekramoddoullah 2006)	0.26	
9	NZPradTrx077717_C01	LP3-1	Implicated in water-stress https://www.uniprot.org/uniprot/Q5G154	0.24	
10	NZPradTrx100333_C02	ASR protein	Involved in sugar and abscisic acid signalling (Çakir et al. 2003)	0.25	0.24
11	NZPradTrx098632_C01	Translation elongation factor-1 alpha	Catalyses the transfer of aminoacylated-tRNAs (Sasikumar et al. 2012)		
12	NZPradTrx098233_C03 ^Na^	Ribulose bisphosphate carboxylase/oxygenase (RuBisCO)	Catalyses carboxylation of RuBP in the first step of the Calvin cycle of photosynthesis (Tabita 1999)		1.57 ^Na^, 0.59 ^Nb^, 0.53 ^Nc^, 0.36 ^Nd^, 0.30 ^Ne^, 0.22 ^Nf^
	NZPradTrx064995_C01 ^Nb^				
	NZPradTrx064875_C01 ^Nc^				
	NZPradTrx098233_C01 ^Nd^				
	NZPradTrx098233_C05 ^Ne^				
	NZPradTrx064875_C02 ^Nf^				
13	NZPradTrx098207_C02 ^Na^	Cysteine proteinase inhibitor CPI-3	Involved in plant development and defence, especially in the regulation of stress responses (Li et al. 2015)		0.77 ^Na^, 0.27 ^Nb^
	NZPradTrx098207_C01 ^Nb^				
14	NZPradTrx105813_C01	PREDICTED: probable fructose-bisphosphate aldolase 2, chloroplastic-like	Plays a key role in glycolysis and gluconeogenesis https://www.uniprot.org/uniprot/Q944G9		0.37
15	NZPradTrx111299_C01 ^Na^	PREDICTED: oxygen-evolving enhancer protein 1, chloroplastic-like isoform 2	Stabilizes the manganese cluster which is the primary site of water splitting https://www.uniprot.org/uniprot/P23321		0.35 ^Na^, 0.32 ^Nb^
	NZPradTrx100425_C01 ^Nb^				
16	NZPradTrx065162_C02	Thiazole biosynthetic enzyme	Thiamine synthesis and DNA damage tolerance (Liu et al. 2015b)		0.34
17	NZPradTrx184720_C01	PSI-D1 precursor	PsaD can form complexes with ferredoxin and ferredoxin-oxidoreductase in photosystem I (PS I) reaction centre. https://www.uniprot.org/uniprot/Q9S7H1		0.22

At T0, 5469 out of the 6312 transcripts (86.6%) were differentially expressed between the needles and the bark. Of these, 3123 were up-regulated in the bark compared to the needles, while 2346 transcripts were up-regulated in the needles. The top 10 most strongly up-regulated transcripts in each of the bark and needles are shown in Table 4 (superscripts are identifiers to help locate the needle (N) or bark (B) transcripts in the ID column of the table). Besides the general function genes and those related with photosynthesis, there was an up-regulation of genes related to terpene ^[B9]^ and lipids biosynthesis ^[B7]^ in the bark and those related to sugars ^[N4]^ and phenolics biosynthesis ^[N1]^ in the needles. Of note is the up-regulation of genes involved in sugar transport in both the needles ^[N3]^ and the bark ^[B2]^, but these are different genes.

**Table 4:** Top 10 up-regulated genes differentially expressed between the bark and needles at T0 (before treatment) for each plant part. The table also shows the ID of the genes assigned in this study for ease of identification in the tables, Scion transcripts code, predicted gene name and function

Part	ID	Scion transcript code	Predicted gene name	Predicted gene function
Bark	B1	NZPradTrx054097_C01	Homeobox transcription factor KN3	Central regulators of meristem cell identity (Guillet-Claude et al. 2004)
	B2	NZPradTrx073079_C03	Transporter, putative	Sugar transport (Weig et al. 1994)
	B3	NZPradTrx087709_C01	Homeobox transcription factor KN1	Central regulators of meristem cell identity (Namroud et al. 2010)
	B4	NZPradTrx055579_C01	Mini zinc finger 1	Regulates several development aspects, including photomorphogenesis, apical dominance, longevity, flower morphology and fertility, as well as root and stem elongation (https://www.uniprot.org/uniprot/Q9CA51)
	B5	NZPradTrx048496_C01	Plastid phosphate translocator	Involved in the exchange of metabolites and inorganic phosphate between stroma and cytosol (Bockwoldt et al. 2019)
	B6	NZPradTrx101882_C01	Auxin-induced protein 5NG4, putative	Transmembrane transporter activity especially during root formation (Busov et al. 2004)
	B7	NZPradTrx103825_C01 NZPradTrx103825_C04	PREDICTED: GDSL esterase/lipase At5g03610-like	Lipid catabolic process (https://www.uniprot.org/uniprot/Q9LZS7)
	B8	NZPradTrx184572_C01	G1-like protein	Polymerizes the backbones of non-cellulosic polysaccharides (hemicelluloses) of plant cell wall https://www.uniprot.org/uniprot/Q570S7
	B9	NZPradTrx055645_C01	PREDICTED: squalene monooxygenase-like	Converts squalene into oxidosqualene, the precursor of all known angiosperm cyclic triterpenoids (Rasbery et al. 2007)
		NZPradTrx096935_C03		
	B10	NZPradTrx093053_C01	Ribulose 1,5-bisphosphate carboxylase/oxygenase small subunit	Catalyses carboxylation of RuBP in the first step of the Calvin cycle of photosynthesis (Tabita 1999)
Needles	N1	NZPradTrx115678_C04	Anthocyanidin reductase	Involved in the biosynthesis of proanthocyanidins (Zhu et al. 2015)
		NZPradTrx115678_C05		
	N2	NZPradTrx090889_C01	Cytochrome P450 CYPA2	Oxidoreductase activity, acting on paired donors, with incorporation or reduction of molecular oxygen https://www.uniprot.org/uniprot/A9F9S4
	N3	NZPradTrx114954_C01	Glucosyltransferase	Transfer of glucose (Chen et al. 2016)
		NZPradTrx086877_C02		
	N4	NZPradTrx088783_C01	Glucose-1-phosphate adenylyltransferase, putative	Involved in the pathway starch biosynthesis (https://www.uniprot.org/uniprot/Q688T8)
	N5	NZPradTrx086324_C01	PREDICTED: LOB domain-containing protein 1-like	Involved in the repression of the homeobox gene BP https://www.uniprot.org/uniprot/Q9FKZ3-1
	N6	NZPradTrx065580_C01	Catalase	Crucial antioxidant enzymes that mitigates oxidative stress to a considerable extent by destroying cellular hydrogen peroxide to produce water and oxygen (Nandi et al. 2019)
	N7	NZPradTrx049683_C01	Photosystem II core complex proteins psbY2C chloroplast precursor	Multi-component pigment-protein complex responsible for water splitting, oxygen evolution, and plastoquinone reduction (Lu 2016)
	N8	NZPradTrx097448_C02	ribonucleoprotein, chloroplast, putative	Involved in chloroplast RNA processing (Tillich et al. 2009)
	N9	NZPradTrx119685_C01	SOUL heme-binding protein	Plays an active role in primary plant metabolic pathways as well as in stress signalling (Shanmugabalaji et al. 2020)
	N10	NZPradTrx184701_C01	chloroplast ribosomal protein S1	Involvement in translation initiation via positioning of initiation mRNA–protein complexes (mRNPs), and the potential involvement of these unique domains in the processivity of chloroplast translation (Manuell et al. 2007)

**Fig. 3:**
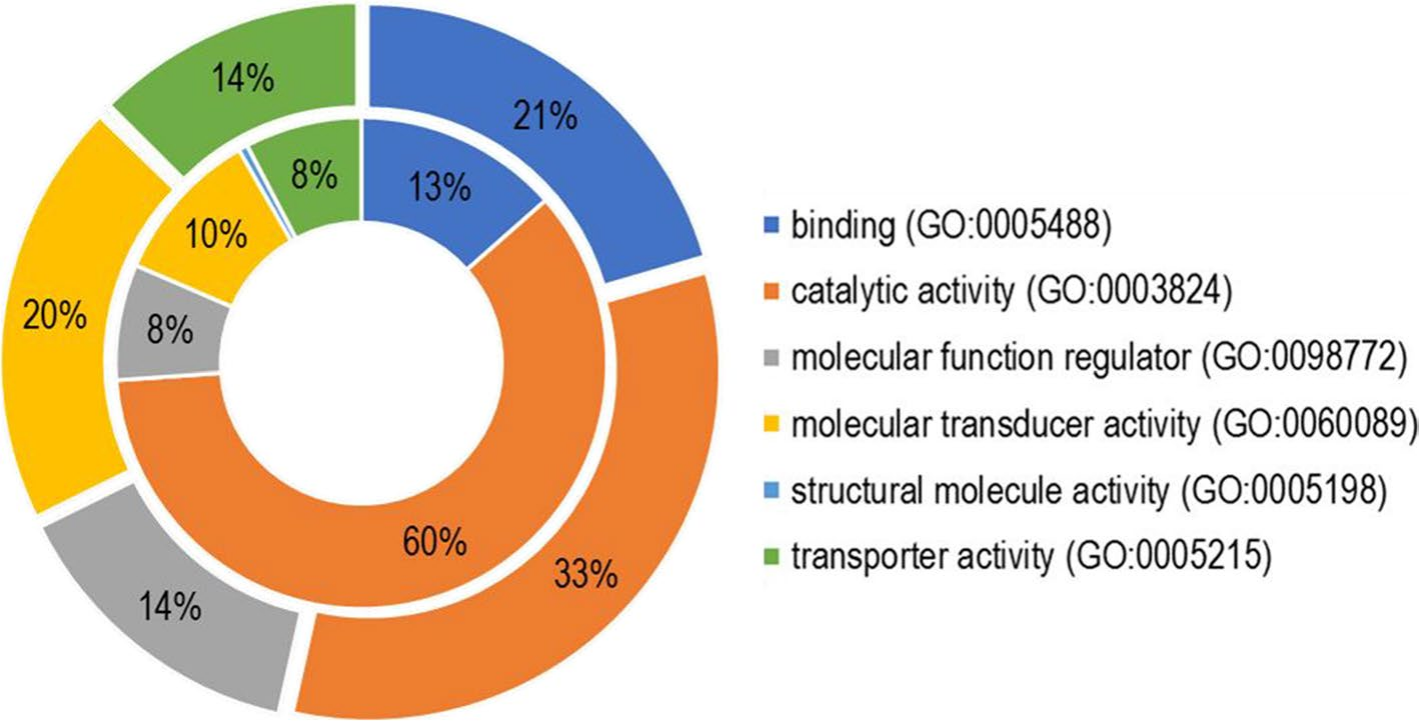
The different molecular functions (GO categories) of the top 100 transcripts that showed up-regulation in the needles when compared with the bark (inner circle) and top 100 transcripts that were up-regulated in the bark when compared with the needles (outer circle) at T0. These up-regulated transcripts represent constitutive responses between plant parts and for each plant part, the percentage of the top 100 upregulated transcripts that were assigned to the GO categories indicated are shown

To assess the overall constitutive functional differences in transcripts differentially upregulated in the needles and the bark, the GO annotation of the top 100 differentially upregulated genes in both plant parts was obtained. There were quantitative differences for all the molecular but not biological or cellular GO categories. In the molecular GO category, a greater proportion of the top upregulated genes in the needles were ascribed to catalytic activity in the needles than in the bark (Fig. 3).

### Overall transcript expression in the needles and the bark after treatment

**Fig. 4:**
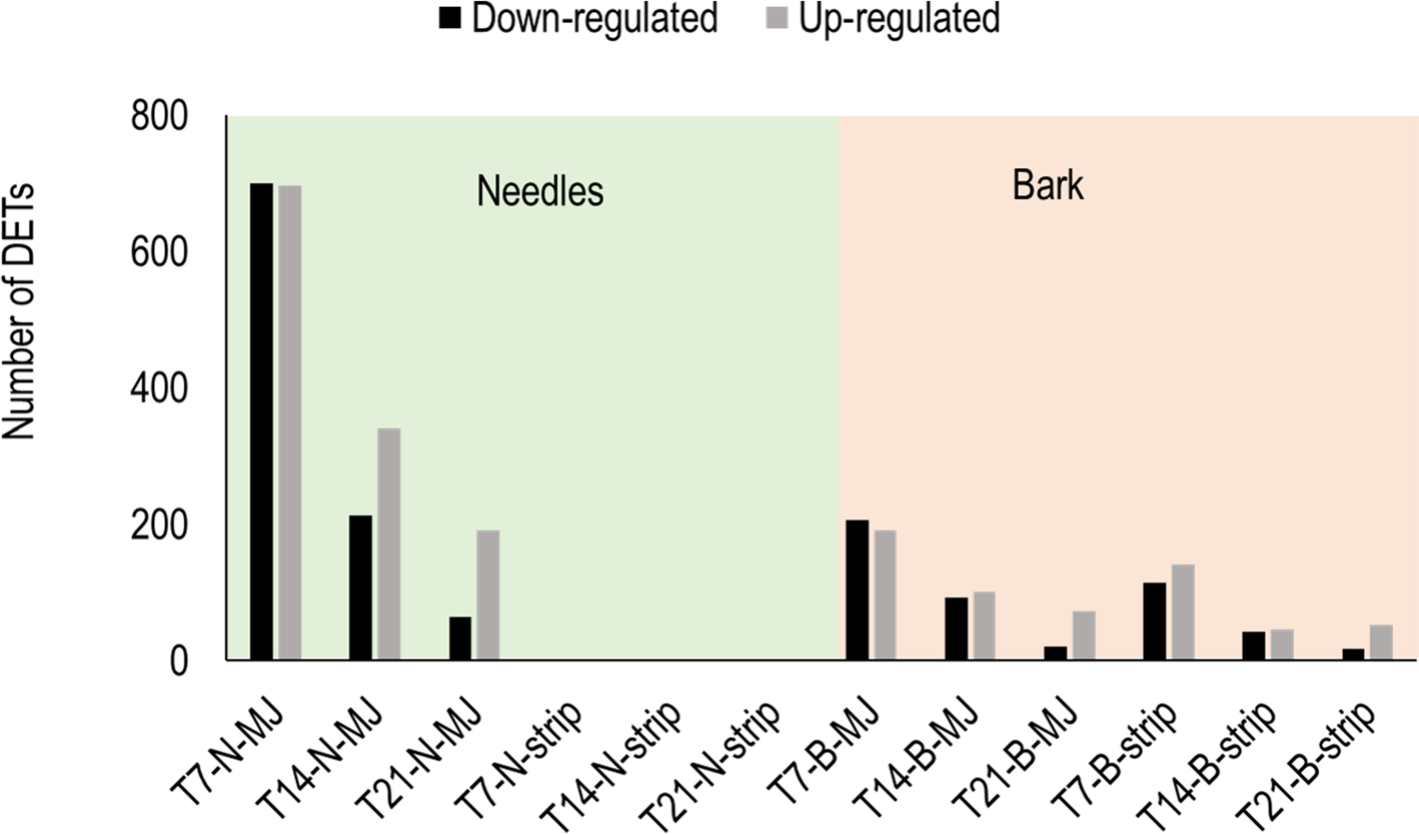
The number of differentially expressed transcripts (DETs) that were up-regulated and down-regulated in *Pinus radiata* needles (N) and bark (B) following methyl jasmonate (MJ) and bark stripping (strip) treatments quantified 7 (T7), 14 (T14) and 21 (T21) days after treatment. No differential expression was detected in the needles following the bark stripping treatment. Note that there could be an overlap in the DETs for different treatments

After treatment, considering all time points, a total of 1479 (23.4%) transcripts were differentially expressed at one time or another. More transcripts responded to treatment in the needles than in the bark and more transcripts were up-regulated than down-regulated (Fig. 4). For both treatments, most differential expression was detected 7 days (T7) after treatment and declined thereafter, although differential expressed transcripts were still evident in both treatments 21 days later (Fig. 4). MJ was applied to both bark and needles and caused more transcript expression than bark stripping in both the needles and the bark (Fig. 4). Indeed, no differential expression of transcripts was detected in the needles following bark stripping. Of the transcripts that were differentially expressed between the bark and needles at T0, only 20% and 1% of those respectively responded following either of the treatments in the bark and needles suggesting that the transcripts that did not differ constitutively (i.e. at T0) between the needles and the bark were more responsive to treatment. One uncharacterised transcript (NZPradTrx091980_C05) that was not present in the transcriptome of untreated samples was present after treatment. One isoform of ribulose bisphosphate carboxylase preprotein (NZPradTrx098233_C06) that is involved in photosynthesis was present before treatment but was missing in all the samples in the bark and the needles after treatment, including the untreated control samples.

**Fig. 5:**
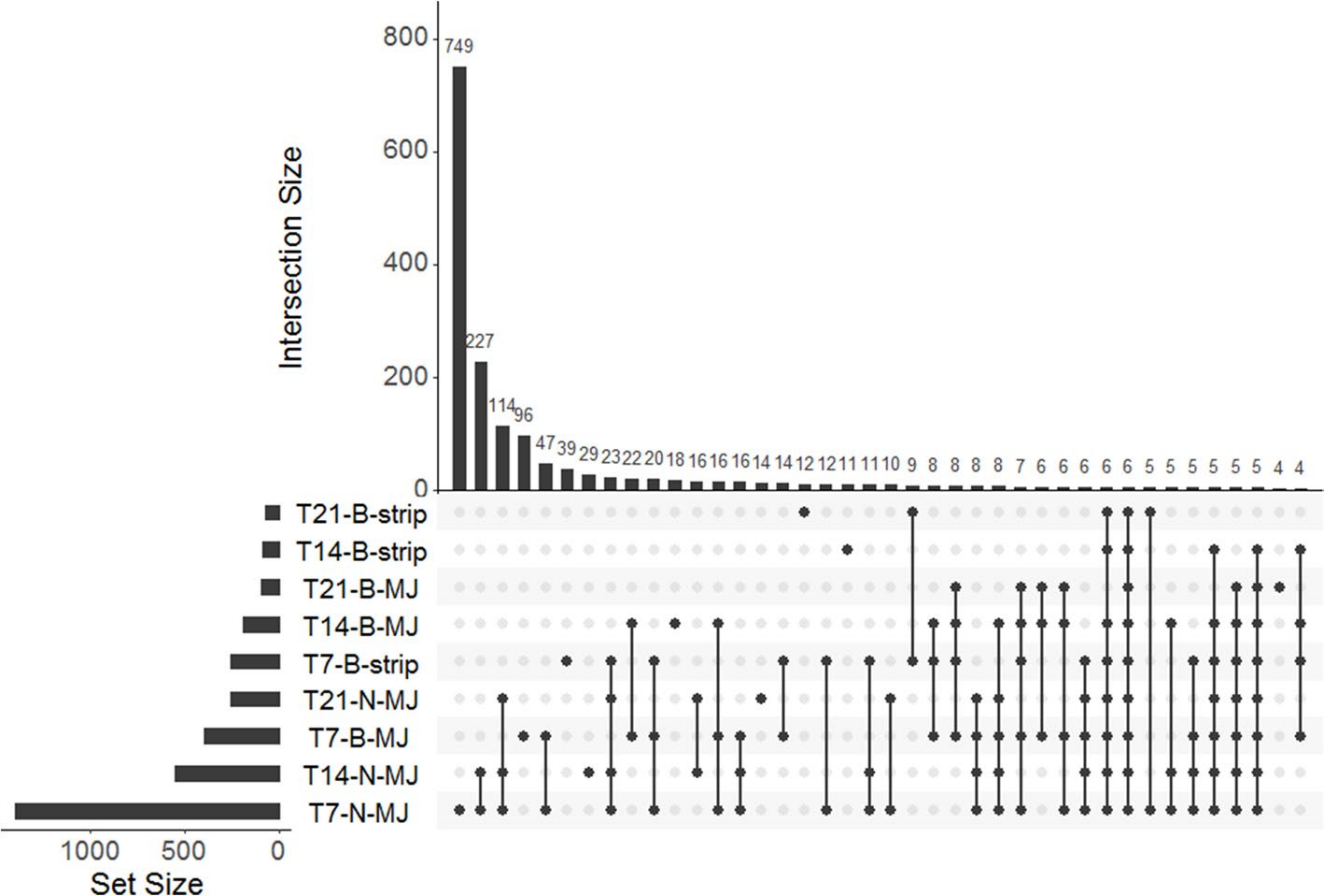
An upset plot showing the number of unique and overlapping differentially expressed transcripts following methyl jasmonate (MJ) and the bark stripping (strip) treatments over time and plant parts (needles [N] and bark [B]). T7, T14 and T21 referred to sampling undertaken 7, 14 and 21 days after treatment respectively. As an example, 749 transcripts in the needles (N) were differentially expressed uniquely at T7 following MJ treatment and were not differentially expressed at any time point in the bark (B) or other time point in the needles (N). Treatments or times where overlapping transcripts occur are linked by lines. For example, the most common overlapping transcripts were the 227 that were differentially expressed only in the needles at T7 and T14 in methyl jasmonate (MJ) treatment. The other transcript combinations are ordered by their frequency of occurrence according to the various unique or overlapping combinations in which they were differentially expressed in the methyl jasmonate (MJ) and bark stripping (strip) treatments at each time. Note that no transcripts were differentially expressed in the needles following bark stripping at any time

Annotations of the top ten genes that were up-regulated or down-regulated for each condition (time × treatment × part) are presented in Table 5. Based on these genes, various functions were detected, indicating that multiple genes are involved in coordinating plant responses to stress. Most of the genes were up-regulated, for example genes associated with primary metabolism, secondary metabolism, digestive inhibitors, pathogenesis-related (PR) protein families, genes involved with physical strengthening of the cell-wall, transcription factors, phytohormones and signalling molecules as well as molecules involved in broad biotic and abiotic stress responses and broad function genes. In contrast, the general catalysts as well as molecules involved in transcription were down-regulated. A subset (968 out of 1479 = 64.7%) of the differentially expressed transcriptome studied was differentially expressed in only one treatment (strip or MJ) (Fig. 5, Table 5). Similarly, non-overlapping differentially expressed transcripts, occurring in only one condition, were detected at different times in the needles and bark (Fig. 5, Table 5).

**Table 5:** Top 10 genes differentially expressed in each of the time periods from T7 to T21 in the bark (B) and needles (N) following bark stripping (S) or methyl jasmonate (MJ) treatment of two-year old *Pinus radiata* plants The Scion transcript code, predicted gene name and predicted functions of the known genes are indicated. Some genes were represented by more than one transcript (isoforms—different Scion *P. radiata* transcript codes that represent one gene in column 1) and multiple copies of an isoform as indicated by the numbers in the parentheses, for example +(2) = two copies of an isoforms relating to the gene were identified, where + = up-regulation, − = down-regulation. The superscript followingnumbers in the parentheses following the gene names represent the core function of the gene among the 11 broad categories listed in the table footnote. For example for the Peptide transporter PTR3-A-like,^a^ the superscript ^a^ denotes that this gene was associated with primary metabolism (see footnote). However, it is recognised that some genes may fall in more than one category. Gene functions are mostly from UniProt [77]

Scion transcript code	Gene name	Function	T7-B-MJ	T7-B-S	T7-N-MJ	T14-B-MJ	T14-B-S	T14-N-MJ	T21-B-MJ	T21-B-S	T21-N-MJ
NZPradTrx081530_C01	Peptide transporter PTR3-A-like^a^	Facilitates amino acid induction (Barnes et al. 1998)	+								
NZPradTrx115883_C01	Granule-bound starch synthase, partial^a^	Responsible for amylose synthesis (Miao et al. 2014)	–								
NZPradTrx113785_C01	GDP-D-mannose-3′,5′-epimerase^a^	Central enzyme of the major ascorbate biosynthesis pathway in higher plants that converts GDP-d-mannose to GDP-l-galactose (Gilbert et al. 2009)	+								
NZPradTrx065162_C02	Thiazole biosynthetic enzyme^b^	Thiamine synthesis and DNA damage tolerance (Liu et al. 2015b)	–								
NZPradTrx083866_C01	1-aminocyclopropane-1-carboxylate oxidase 3^g^	Production of ethylene, that functions as a mediator of responses to external stimuli, such as wounding (Houben and Van de Poel 2019)	+	+		+	+				
NZPradTrx117447_C01	PREDICTED: transcription factor bHLH126-like^f^	Transcription factors play a central role in a number of biological processes, producing, for example, the induction of specific genes in response to particular stimuli as well as controlling the cell type specific or developmentally regulated expression of other genes (Latchman 2008)	+ (2)			+(2)			+(3)	+	
NZPradTrx117447_C02											
NZPradTrx091619_C02											
NZPradTrx113021_C04	Cytochrome P450 CYPC^h^	Key players in plant development and defence (Xu et al. 2015)	-(2)			–					
NZPradTrx117482_C10											
NZPradTrx103647_C02	Oleoyl-acyl carrier protein thioesterase, partial^a^	Plays an essential role in chain termination during de novo fatty acid synthesis https://www.uniprot.org/uniprot/Q42561	–								
NZPradTrx111880_C01	Cell wall invertase^a^	Mediates reduced export of sucrose or enhanced import of hexoses at the site of infection (Proels and Hückelhoven 2014) [78]	+				+			+ (2)	
NZPradTrx132560_C01											
NZPradTrx186688_C01	DNA binding protein, putative^i^	DNA binding proteins serve two principal functions: to organize and compact the chromosomal DNA and to regulate and effect the processes of transcription, DNA replication, and DNA recombination (Travers 2001).		+		–		–			
NZPradTrx187077_C01											
NZPradTrx065807_C02	PREDICTED: cleavage and polyadenylation specificity factor subunit 5-like^i^	Component of the cleavage factor Im (CFIm) complex that functions as an activator of the pre-mRNA 3′-end cleavage and polyadenylation processing required for the maturation of pre-mRNA into functional mRNAs https://www.uniprot.org/uniprot/Q16630		–							
NZPradTrx095732_C01	Thaumatin-like protein^d^	Involved in local responses of roots to colonization by non-pathogenic plant growth-promoting rhizobacteria (PGPR) fluorescent Pseudomonas spp. (Léon-Kloosterziel et al. 2005)	–	-,+ (2)			+		+	+	
NZPradTrx064724_C01											
NZPradTrx108940_C08											
NZPradTrx087317_C02											
NZPradTrx038584_C01	Chloroplast threonine deaminase 1 precursor^c^	Useful in isoleucine (Ile) biosynthesis and impairing digestive processes in the insect gut (Chen et al. 2007)		+	+		+	+		+	+
NZPradTrx111230_C01	Triacylglycerol lipase, putative^a^	Releases fatty acids from a number of different substrates (Padham et al. 2007)		–							
NZPradTrx084103_C02	PREDICTED: glutamate--cysteine ligase, chloroplastic-like^d^	Seems to play an important role in controlling the expression of resistance responses like the regulation of salicylic acid (SA) and phytoalexin (camalexin) production. Involved in resistance to fungal and bacterial pathogens. https://www.uniprot.org/uniprot/P46309		+							
NZPradTrx074370_C02, NZPradTrx132647_C01	PREDICTED: lysine histidine transporter 2-like^k^	Amino acid-proton symporter. Transporter with a broad specificity for neutral and acidic amino acids https://www.uniprot.org/uniprot/Q9LRB5		+			+		+		+(2)
NZPradTrx098051_C01	PREDICTED: endo-1,3;1,4-beta-D-glucanase-like^j^	Implicated in responses to stress, wounding, and pathogen infection (Rezzonico et al. 1998)		+							
NZPradTrx053937_C01	2-methyl-6-phytylbenzoquinone methyltranferase^j^	One of the regulators of the composition of tocopherols-class of compounds that function as lipid soluble antioxidants that are extremely potent quenchers of singlet oxygen and free radical species (Shintani et al. 2002)		–							
NZPradTrx119228_C01	4-hydroxyphenyl-pyruvate dioxygenase^a^	Plays an important role in degrading aromatic amino acids (Fritze et al. 2004)			+			+			
NZPradTrx184501_C01	PREDICTED: 50S ribosomal protein L6, chloroplastic-like^i^	Binds directly to 23S ribosomal RNA and is located at the aminoacyl-tRNA binding site of the peptidyltransferase centre. https://www.uniprot.org/uniprot/O23049			–						
NZPradTrx186075_C01	PREDICTED: hexokinase-1-like^a^	Fructose and glucose phosphorylating enzyme https://www.uniprot.org/uniprot/Q42525			–			–			
NZPradTrx105399_C03	PREDICTED: leucine-rich repeat-containing protein 40-like^j^	Plays crucial roles in development and stress responses (Liu et al. 2017) [17, 79]			–						
NZPradTrx051602_C02	Sodium-bile acid cotransporter, putative^k^	Is involved in photorespiratory metabolism (South et al. 2017)			–						
NZPradTrx082621_C01	Mitogen activated protein kinase 6^j^	Involved in oxidative stress-mediated signalling cascade (such as ozone) https://www.uniprot.org/uniprot/Q39026			+						
NZPradTrx033779_C01	PREDICTED: pentatricopeptide repeat-containing protein At1g62670, mitochondrial-like^i^	Binds one or several organellar transcripts, and influences their expression by altering RNA sequence, turnover, processing, or translation (Barkan and Small 2014)			–						
NZPradTrx184660_C01	PREDICTED: PGR5-like protein 1A, chloroplastic-like^a^	Ferredoxin-plastoquinone reductase involved in cyclic electron flow (CEF) around photosystem I https://www.uniprot.org/uniprot/Q8H112			–						
NZPradTrx097586_C01	Type III chlorophyll a /b-binding protein^a^	Functions as a light receptor, capturing and delivering excitation energy to photosystems with which it is closely associated https://www.uniprot.org/uniprot/P27523			–						
NZPradTrx101698_C02	PrMC3^b^	Predicted to encode a chalcone-synthase-like protein (Walden et al. 1999)				–			–		
NZPradTrx117804_C07	PREDICTED: probable carboxylesterase 2^a^	Carboxylesterases hydrolyse esters of short-chain fatty acids (Marshall et al. 2003)				–					
NZPradTrx100227_C01	PREDICTED: medium-chain-fatty-acid--CoA ligase^a^	Catalyses the esterification, concomitant with transport, of exogenous fatty acids into metabolically active CoA thioesters for subsequent degradation or incorporation into phospholipids https://www.uniprot.org/uniprot/P38135				+					
NZPradTrx081530_C01	PREDICTED: peptide transporter PTR3-A-like^a^	Facilitates amino acid induction (Barnes et al. 1998)				+					
NZPradTrx192941_C01	Beta-amylase	Involved in starch breakdown in plants (Kaplan and Guy 2004)				+					
NZPradTrx052040_C01	PREDICTED: oleosin 16 kDa-like^j^	May have a structural role to stabilize the lipid body during desiccation of the seed by preventing coalescence of the oil. https://www.uniprot.org/uniprot/Q42980				–					
NZPradTrx108711_C04	PREDICTED: putative UDP-rhamnose:rhamnosyltransferase 1-like^a^	Involved in fatty acid metabolism (van der Sluis and Erasmus 2016)					+				
NZPradTrx112833_C08	Tify domain containing protein^i^	Found in a variety of plant transcription factors https://pfam.xfam.org/family/PF06200					+		+		+
NZPradTrx112833_C07											
NZPradTrx071306_C02	PREDICTED: transmembrane ascorbate ferrireductase 1-like^j^	Catalyses ascorbate-dependent trans-membrane ferric-chelate reduction https://www.uniprot.org/uniprot/Q8L856					+				
NZPradTrx051982_C01	PREDICTED: histone H2B.2-like isoform 2^i^	Play a central role in transcription regulation, DNA repair, DNA replication and chromosomal stability https://www.uniprot.org/uniprot/Q5QNW6					–				
NZPradTrx119456_C01	PR10-1.13^j^	Involved in defence against pathogen infection and other environmental stresses (Liu et al. 2005)					+				
NZPradTrx053878_C02	Aldehyde dehydrogenase^a^	Involved in plant metabolism and contributes to aldehyde homeostasis to eliminate toxic aldehydes (Zhao et al. 2017)						+(3)			+(3)
NZPradTrx053878_C01											
NZPradTrx053878_C03											
NZPradTrx087148_C01	PREDICTED: lanC-like protein 2-like^g^	May play a role in abscisic acid (ABA) signalling https://www.uniprot.org/uniprot/F4IEM5						+			
NZPradTrx115807_C06	Hydrolase, putative^j^	Enzyme which catalyses hydrolysis reaction, i.e. the addition of the hydrogen and hydroxyl ions of water to a molecule with its consequent splitting into two or more simpler molecules. https://www.uniprot.org/keywords/KW-0378						+			+
NZPradTrx112951_C03	Embryo-abundant protein^j^	May act as a cytoplasm protectant during desiccation. https://www.uniprot.org/uniprot/P46520						+			
NZPradTrx097637_C01	PREDICTED: leucoanthocyanidin dioxygenase-like^b^	Involved in anthocyanin and protoanthocyanidin biosynthesis by catalysing the oxidation of leucoanthocyanidins into anthocyanidins https://www.uniprot.org/uniprot/Q96323						+			
NZPradTrx112166_C01	Peroxidase-like protein, partial^j^	Response to oxidative stress https://www.uniprot.org/uniprot/Q24925						+			+
NZPradTrx082621_C01	Mitogen activated protein kinase 6^g^	Plays key role in the transduction of environmental and developmental signals through phosphorylation of downstream signalling targets (Jagodzik et al. 2018)						+			
NZPradTrx110107_C07	PREDICTED: transcription factor aborted microspores-like	Required for male fertility and pollen differentiation, especially during the post-meiotic transcriptional regulation of microspore development within the developing anther https://www.uniprot.org/uniprot/Q9ZVX2							+		
NZPradTrx112236_C02	Laccase^b^	Involved in phenolic metabolism and functioning of cell wall (Ranocha et al. 2002)							+		
NZPradTrx089433_C01	Lipoxygenase 2^b^	Essential for formation of green leaf volatiles and five-carbon volatiles (Mochizuki et al. 2016)							+		
NZPradTrx109272_C04	Malic enzyme, putative^a^	Catalyses the oxidative decarboxylation of malate to form pyruvate, a reaction important in a number of metabolic pathways (Zhang et al. 2016)							–	–	
NZPradTrx107808_C01	Putative flavoprotein-containing polyamine oxidase, partial^b^	Involved in drought stress response and flavonoid biosynthesis (Kamada-Nobusada et al. 2008)							+		
NZPradTrx049513_C01	Putative proline-rich arabinogalactan protein 4^e^	Contributes to the strengthening of cell walls of quickly growing organs (Hijazi et al. 2014)								+	
NZPradTrx049513_C02											
NZPradTrx079868_C01	PREDICTED: (RS)-norcoclaurine 6-O-methyltransferase-like^b^	Involved in the biosynthesis of (S)-coclaurine, the common precursor of all benzylisoquinoline alkaloids. https://www.uniprot.org/uniprot/Q6WUC1								–	
NZPradTrx054832_C01	Aquaporin-like protein^k^	Involved in transport of water and other small neutral molecules across cellular biological membranes (Kapilan et al. 2018)								+	
NZPradTrx069597_C01	Acetyl-CoA carboxylase BCCP subunit^a^	Catalyses the first committed step of fatty acid synthesis, the carboxylation of acetyl-CoA to malonyl-CoA (Sasaki and Nagano 2004)								–	
NZPradTrx117954_C05	E-alpha-bisabolene synthase^b^	Involved in defensive oleoresin formation in conifers in response to insect attack or other injury. Involved in sesquiterpene (C15) olefins biosynthesis https://www.uniprot.org/uniprot/O81086								+	
NZPradTrx087252_C01	TPA: putative GID1-like gibberellin receptor^g^	Involved in gibberellin signalling (Sun 2011)									+
NZPradTrx074370_C01	Putative proline transporter^k^	Mediates the amino acid proline and glycine betaine transport https://www.uniprot.org/uniprot/P92961									+(2)
NZPradTrx112166_C01											
NZPradTrx113904_C06/ NZPradTrx101343_C01	PREDICTED: clavaminate synthase-like protein At3g21360-like^j^	Associated with metal ion binding and oxido-reductase activity https://www.uniprot.org/uniprot/Q9LIG0									+

^a^primary metabolism

^b^secondary metabolism

^c^digestive inhibitors

^d^pathogenesis-related (PR) protein families

^e^genes involved with physical strengthening of the cell-wall

^f^transcription factors

^g^phytohormones and signalling molecules

^h^general catalysts

^i^molecules involved in transcription

^j^molecules involved in broad biotic and abiotic stress responses

^k^broad function genes

### Gene expression after MJ treatment

A stronger response to the MJ treatment was detected in the needles than the bark, where 2206 versus 683 out of 6312 transcripts studied were differentially expressed, respectively (Fig. 4). Annotations of the non-overlapping, differentially expressed transcripts showed that MJ caused the unique differential expression of more genes that are directly involved in the metabolism of sugars, fatty acids and amino acids in both the bark and the needles compared with the bark stripping (Table 6).

**Table 6:** Number of differentially expressed (DETs) transcripts (up to a maximum of top10) that were unique (non-overlapping) for each condition (time × treatment × plant part) category. The table also shows the ID of the genes assigned in this study for ease of identification in the tables, Scion transcripts code, predicted gene name and function. These transcripts were not expressed at any other time or treatment. T7, T14 and T21 represents respectively 7, 14 and 21 days after application of methyl jasmonate (MJ) and bark strip (strip) treatments in the bark (B) or needles (N). (+) = up-regulated and (−) = down-regulated. Only transcripts with predicted gene functions are included. The predicted gene functions are mostly from UniProt [77]

Condition	No. unique DETs	ID	*P. radiata* code	Gene name	Predicted gene function	Direction
T7-B-MJ	96	U1	NZPradTrx115883_C02	granule bound starch synthase 1a precursor	Involved in the pathway starch biosynthesis https://www.uniprot.org/uniprot/P0C585	–
		U2	NZPradTrx184661_C01	PREDICTED: putative caffeoyl-CoA O-methyltransferase At1g67980-like	Involved in the reinforcement of the plant cell wall. Also involved in the responding to wounding or pathogen challenge by the increased formation of cell wall-bound ferulic acid polymers https://www.uniprot.org/uniprot/Q9C9W3	–
		U3	NZPradTrx108036_C04	Cytochrome b reductase	Required for the NADH-dependent electron transfer involved in the desaturation and hydroxylation of fatty acids and in the desaturation of sterol precursors https://www.uniprot.org/uniprot/Q9ZNT1	–
		U4	NZPradTrx119186_C01	DEAD-box RNA helicase	Ubiquitous in RNA-mediated processes and function by coupling cycles of ATP binding and hydrolysis to changes in affinity for single-stranded RNA https://www.ncbi.nlm.nih.gov/pmc/articles/PMC3032546/	–
		U6	NZPradTrx060156_C02	PREDICTED: probable rhamnose biosynthetic enzyme 1	Involved with nucleotide-sugar metabolic process https://www.uniprot.org/uniprot/A0A1U7W8H4	+
		U7	NZPradTrx119948_C01	PREDICTED: protein HOTHEAD-like	Required to limit cellular interactions between contacting epidermal cells during floral development (Krolikowski et al. 2003)	+
		U8	NZPradTrx119070_C01	PREDICTED: mitochondrial-processing peptidase subunit alpha-like	Substrate recognition and binding subunit of the essential mitochondrial processing protease (MPP), which cleaves the mitochondrial sequence off newly imported precursors proteins. https://www.uniprot.org/uniprot/P29677	+
		U9	NZPradTrx110606_C03 NZPradTrx110606_C04	snakin	Active against fungal and bacterial plant pathogens (Berrocal-Lobo et al. 2002)	–
		U10	NZPradTrx094750_C01	PREDICTED: zinc finger CCCH domain-containing protein 20-like	Known to play important roles in RNA processing as RNA-binding proteins in animals (Wang et al. 2008)	–
T7-B-strip	39	U11	NZPradTrx111276_C02	low molecular weight heat-shock protein	Expressed in plants experiencing high-temperature stress (Hernandez and Vierling 1993)	–
		U12	NZPradTrx109179_C02	LP3-1	Shown to be up-regulated in response to water deficit stress and to also act as transcription factors for genes likely involved in hexose transport (Lecoy and García-Gil 2020)	–
			NZPradTrx077717_C01			
		U13	NZPradTrx112152_C04	PREDICTED: L-type lectin-domain containing receptor kinase IV.1-like	Involved in resistance response to the pathogenic oomycetes, promotes hydrogen peroxide production and cell death https://www.uniprot.org/uniprot/Q9LXA5	+
		U14	NZPradTrx082734_C01	Casparian strip domain-like gene	Recruit the lignin polymerisation machinery necessary for the deposition of Casparian strips in the endodermis https://www.ebi.ac.uk/interpro/entry/InterPro/IPR006459/	–
		U15	NZPradTrx105759_C05	Methyl esterase 13	Involved in jasmonic and salicylic acid metabolic process https://www.uniprot.org/uniprot/F4IE65	+
		U16	NZPradTrx042090_C01	Geranyl diphosphate synthase	Catalyses the condensation of dimethylallyl diphosphate and isopentenyl diphosphate to geranyl diphosphate, the key precursor of monoterpene biosynthesis (Burke et al. 1999)	+
		U17	NZPradTrx064702_C01	Class II chitinase	Involved in the defence response against pathogen and fungal infection (de A. Gerhardt et al. 1997)	–
		U18	NZPradTrx105720_C01	Defensin	Inhibits the growth of a broad range of pathogens, including bacteria, fungi and viruses (Ermakova et al. 2016; Picart et al. 2012) [75, 76].	–
		U19	NZPradTrx119059_C01	Annexin p33	Central regulator or effector of plant growth and stress signalling (Mortimer et al. 2008)	–
		U20	NZPradTrx118949_C01	Peroxiredoxin	Guardian against oxidative stress and modulator of peroxide signalling (Perkins et al. 2015)	–
T7-N-MJ	751	U21	NZPradTrx110565_C01	UDP-sulfoquinovose synthase	Involved in the biosynthesis of sulfolipids found in thylakoid membranes. Converts UDP-glucose and sulfite to the sulfolipid head group precursor UDP-sulfoquinovose https://www.uniprot.org/uniprot/O48917	–
		U22	NZPradTrx064995_C02	Chloroplast ribulose bisphosphate carboxylase/oxygenase activase alpha1, partial	Catalyses carboxylation of RuBP in the first step of the Calvin cycle of photosynthesis (Tabita 1999)	–
		U23	NZPradTrx088104_C02	RNA polymerase sigma factor rpoD, putative	Initiation factor that promotes the attachment of RNA polymerase to specific initiation sites https://www.uniprot.org/uniprot/P00579	–
		U24	NZPradTrx081803_C01	PREDICTED: mitochondrial carnitine/acylcarnitine carrier-like protein-like	Mediates the transport of acylcarnitines of different length across the mitochondrial inner membrane from the cytosol to the mitochondrial matrix for their oxidation by the mitochondrial fatty acid-oxidation pathway https://www.uniprot.org/uniprot/O43772	–
		U25	NZPradTrx086144_C02	Chloroplast omega-6 fatty acid desaturase	Introduces the second double bond in the biosynthesis of 16:3 and 18:3 fatty acids, important constituents of plant membranes. It is thought to use ferredoxin as an electron donor and to act on fatty acids esterified to galactolipids, sulfolipids and phosphatidylglycerol https://www.uniprot.org/uniprot/P46312	–
		U26	NZPradTrx065194_C01	Glutamate--ammonia ligase	Key enzyme of ammonium assimilation and recycling in plants where it catalyses the synthesis of glutamine from ammonium and glutamate (Lothier et al. 2011)	–
		U27	NZPradTrx077590_C01	PREDICTED: ATP synthase gamma chain, chloroplastic-like	Produces ATP from ADP in the presence of a proton gradient across the membrane. The gamma chain is believed to be important in regulating ATPase activity and the flow of protons through the CF_0_ complex https://www.uniprot.org/uniprot/Q01908	–
		U28	NZPradTrx064646_C01	PREDICTED: photosystem I reaction center subunit XI, chloroplastic-like	Involved in photosynthesis https://www.uniprot.org/uniprot/Q41385	–
		U29	NZPradTrx115121_C05	glutathione peroxidase-like protein, partial	Protects cells from phospholipid hydroperoxides and nonphospholipid peroxides during oxidative stress https://www.uniprot.org/uniprot/P36014	+
		U30	NZPradTrx186664_C01	F353614_1 senescence-associated protein SPA15	May be involved in the regulation of leaf senescence https://www.uniprot.org/uniprot/Q65XF2	–
T14-B-MJ	18	U31	NZPradTrx192941_C01	Beta-amylase	Involved in starch breakdown in plants (Kaplan and Guy 2004)	+
		U32	NZPradTrx076831_C01	UV-B receptor 1	Involved in response to UV-B (Loyola et al. 2016)	+
		U33	NZPradTrx044917_C01	Putative cyclophilin	Involved in various physiological processes including transcriptional regulation, organogenesis, photosynthetic and hormone signalling pathways, stress adaptation and defence responses (Barbosa dos Santos and Park 2019)	–
		U34	NZPradTrx119079_C01	Xyloglucan endotransglucosylase/hydrolase 13	Cleaves xyloglucan polymers, an essential constituent of the primary cell wall, and thereby participates in cell wall construction of growing tissues. https://www.uniprot.org/uniprot/Q9FKL8	–
		U35	NZPradTrx037564_C01	PREDICTED: bidirectional sugar transporter SWEET3-like	Mediates both low-affinity uptake and efflux of sugar across the plasma membrane https://www.uniprot.org/uniprot/Q6NQN5	–
		U36	NZPradTrx118938_C01	Glycine-rich RNA-binding protein	Plays a role in RNA transcription or processing during stress. Binds RNAs and DNAs sequence with a preference to single-stranded nucleic acids. https://www.uniprot.org/uniprot/Q03250	–
		U37	NZPradTrx109658_C01	Probable aquaporin	Involved in transport of water and other small neutral molecules across cellular biological membranes (Kapilan et al. 2018)	–
		U38	NZPradTrx094541_C02	PREDICTED: methionine gamma-lyase-like	Involved in amino acid catabolism (Ravanel et al. 1998)	+
T14-B-strip	12	U39	NZPradTrx119456_C01	PR10-1.13	Involved in defence against pathogen infection and other environmental stresses (Liu et al. 2005)	+
		U40	NZPradTrx098320_C05	PREDICTED: LOB domain-containing protein 1-like	Controls the proximal-distal patterning in petals and the adaxial-abaxial determination of leaves. Involved in the repression of the homeobox gene BP https://www.uniprot.org/uniprot/Q9FKZ3-1	+
		U41	NZPradTrx073494_C01	TPA: putative ARF GTPase-activating domain family protein	Have potential roles in cell migration, central to normal physiology in embryogenesis, the inflammatory response and wound healing (Campa and Randazzo 2008)	–
		U42	NZPradTrx103835_C01	2C-methyl-D-erythritol 2,4-cyclodiphosphate synthase	Involved in the biosynthesis of isopentenyl diphosphate (IPP) and dimethylallyl diphosphate (DMAPP), two major building blocks of terpenoid compounds https://www.uniprot.org/uniprot/P62617	+
		U43	NZPradTrx102746_C02	S6 ribosomal protein	Key downstream effector of the target of rapamycin (TOR) kinase pathway that regulates various biological processes, including translation, synthesis of ribosomal proteins, and transcription of rRNA (Kim et al. 2014)	+
		U44	NZPradTrx094959_C01	Pathogenesis-related protein 10	Involved in a cell wall rigidification to signal transduction and antimicrobial activity (Liu and Ekramoddoullah 2006)	+
		U45	NZPradTrx096309_C03	Dirigent-like protein	Predominant roles in defence responses, secondary metabolism, and fiber biosynthesis (Li et al. 2017)	+
		U46	NZPradTrx105315_C01	PREDICTED: uncharacterized LOC101219508		–
		U47	NZPradTrx077043_C01	FAD/NAD(P)-binding oxidoreductase domain-containing protein	Catalyses a wide variety of redox reactions with many different substrates (Sellés Vidal et al. 2018)	–
		U48	NZPradTrx110593_C01	PREDICTED: chaperonin CPN60-2, mitochondrial-like	Implicated in mitochondrial protein import and macromolecular assembly. May facilitate the correct folding of imported proteins. May also prevent misfolding and promote the refolding and proper assembly of unfolded polypeptides generated under stress conditions in the mitochondrial matrix. https://www.uniprot.org/uniprot/Q05046	+
T14-N-MJ	30	U49	NZPradTrx118421_C03	Caffeic acid O-methyltransferase	Catalyses the conversion of caffeic acid to ferulic acid and of 5-hydroxyferulic acid to sinapic acid. The resulting products may subsequently be converted to the corresponding alcohols that are incorporated into lignins https://www.uniprot.org/uniprot/Q06509	+
		U50	NZPradTrx079649_C05	Geranyl diphosphate synthase	Catalyses the condensation of dimethylallyl diphosphate and isopentenyl diphosphate to geranyl diphosphate, the key precursor of monoterpene biosynthesis (Burke et al. 1999)	+
			NZPradTrx079649_C03			
			NZPradTrx079649_C02			
		U51	NZPradTrx122822_C01	PREDICTED: F-box protein GID2-like	Essential component of the SCF-type E3 ligase complex, SCF(GID2), a complex that positively regulates the gibberellin signalling pathway https://www.uniprot.org/uniprot/Q9STX3	+
		U52	NZPradTrx083848_C01	Chlorophyllase	The first enzyme involved in chlorophyll (Chl) degradation and catalyses the hydrolysis of ester bond to yield chlorophyllide and phytol (Tsuchiya et al. 1999)	+
		U53	NZPradTrx103321_C01	Phenylalanine ammonia-lyase	Phenylalanine aminomutase that catalyses the rearrangement of L-phenylalanine to R-beta-phenylalanine. Catalyses the first committed step in the biosynthesis of the side chain of the alkaloid taxol (paclitaxel) https://www.uniprot.org/uniprot/Q68G84	+
		U54	NZPradTrx071573_C01	Starch synthase isoform II	Contributes to the extension of glucan chains in the synthesis of starch (Edwards et al. 1999)	+
		U55	NZPradTrx105898_C01	Glutamate-1-semialdehyde 2,1-aminomutase	Essential enzyme in the pathway that leads to the synthesis of chlorophyll and other tetrapyrroles in plants and some bacteria (Tyacke et al. 1995)	–
		U56	NZPradTrx182827_C01	PREDICTED: LRR receptor-like serine/threonine-protein kinase FLS2-like	Constitutes the pattern-recognition receptor (PPR) that determines the specific perception of flagellin (flg22), a potent elicitor of the defence response to pathogen-associated molecular patterns (PAMPs) https://www.uniprot.org/uniprot/Q9FL28	+
		U57	NZPradTrx184681_C01	FK506 binding-like protein	Involved in diverse cellular functions including protein folding, cellular signalling, apoptosis and transcription (Tong and Jiang 2016)	+
		U58	NZPradTrx094486_C01	Putative UDP-glucose:flavonoid glucosyltransferase	Enhances the solubility of anthocyanins (Chen et al. 2011)	+
T21-B-MJ	4	U59	NZPradTrx083714_C01	PREDICTED: protein GLUTAMINE DUMPER 1-like	Involved in the regulation of amino acid metabolism, in the salicylic acid (SA) pathway and in the geminivirus-host interaction https://www.uniprot.org/uniprot/O81775	+
		U60	NZPradTrx053990_C01	PREDICTED: cytochrome P450 71A1-like	Involved in the metabolism of compounds associated with the development of flavour in the ripening fruit process, possibly by acting as trans-cinnamic acid 4-hydrolase https://www.uniprot.org/uniprot/P24465	+
		U61	NZPradTrx105443_C01	GMP synthase [glutamine-hydrolyzing] subunit A, putative	Involved in de novo biosynthesis of guanosine nucleotides https://www.brenda-enzymes.org/enzyme.php?ecno=6.3.5.2	+
		U62	NZPradTrx112236_C01	Laccase	Involved in phenolic metabolism and functioning of cell wall (Ranocha et al. 2002)	+
T21-B-S	13	U63	NZPradTrx087634_C02	Properoxidase	Involved in lignification, cell elongation, stress defence and seed germination (Shigeto and Tsutsumi 2016)	+
		U64	NZPradTrx103699_C01	Oxidoreductase, 2OG-Fe(II) oxygenase family protein	Involved in defence against pathogens (Van Damme et al. 2008)	+

Six transcripts were consistently differentially expressed from T7 – T21 (Fig. 5) in the methyl jasmonate-induced transcriptome of the bark (B-MJ) and these were mostly up-regulated. Annotations of these transcripts showed that the genes were mostly involved in generating energy from various substrates, particularly glucose and fatty acids. In the needles treated with methyl jasmonate (N-MJ), 114 transcripts were consistently differentially expressed from T7 - T21 (Fig. 5). These genes were mostly directly associated with defence as well as chemical and physical structures, for example those involved in phenolic biosynthesis and structural components of the cell wall (Table 5).

### Gene expression after bark stripping

Bark stripping did not cause any systemic response in the needles at any time point (Fig. 4). The strip induced bark transcriptome had, among the top genes, those involved in defence against pathogens, such as chitinases^[U17]^, PR10^[U39]^ and defensins^[U18]^. Bark stripping also caused differential expression of water-stress responsive genes^[U12,U39]^ as well as genes related to replacement of tissues^[U34]^ (Table 6). The difference in the representation of genes is likely related to the kind of damage incurred by the two stressors.

Both stressors caused differential expression of genes related to secondary metabolism (Table 5), including metabolism of monoterpenes (e.g. geranyl diphosphate synthase), phenolics (e.g. laccases) and alkaloids (e.g. phenylalanine ammonia-lyase). The differential expression of genes associated with lignification of cell walls were also identified for both treatments in the needles and the bark, emphasising the role of cell wall physical properties in stress responses. For some genes, the same gene was represented by different isomorphs in the different conditions such as geranyl diphosphate synthase in B-strip and N-MJ treatment/part combinations shown in Table 5. Only 6 differentially expressed genes were consistently differentially expressed following both treatments across all times and plant parts, except that no differential expression occurred in the needles following the strip treatment. Annotations of these transcripts mostly showed genes related to amino acid synthesis.

#### Time progression of genes

**Fig. 6:**
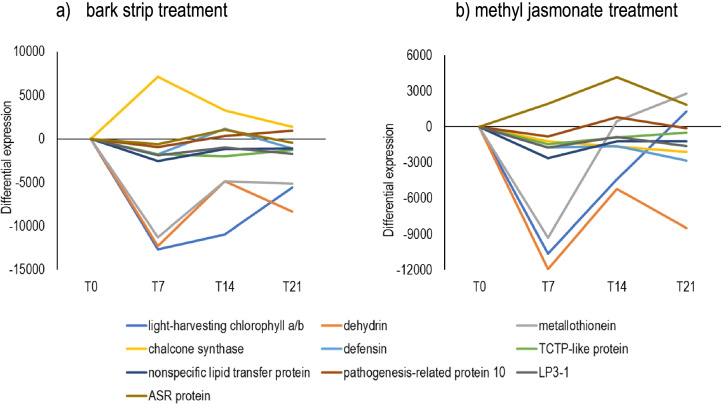
Time progression in the differential expression (control versus treatment) of the top 10 most expressed genes in the constitutive transcriptome of *Pinus radiata*. The genes are detailed in Table 3 and their differential expression in bark is shown following **a** bark strip and **b** methyl jasmonate treatments. The average change in expression was estimated at each time point by comparing the raw counts for the bark strip or methyl jasmonate induced transcripts and the transcripts from control treatments (mean of treatment – mean of control) for a specific time and were adjusted according to the differences in basal expression of the treatment groups at T0. T0 is before treatment applications and T7, T14 and T21 correspond to 7, 14 and 21 days after treatment application, respectively

Not only did the treatments differ in the magnitude of their general response through time (Figs. 1, 4 and 5), but the pattern of response of individual genes differed between treatments. For the top ten expressed transcripts in the constitutive transcriptome (assessed at T0) of the bark and the needles (ID numbers 1 to 10 in Table 3), Fig. 6 shows the time progression of differential expression following stripping and methyl jasmonate application.

There was a tendency for genes to be up-regulated or down-regulated following both treatments. Of the three genes (dehydrin, light-harvesting chlorophyll a/b-binding polypeptide and metallothionein) that showed marked down-regulation, only dehydrin showed significant down-regulation at T7 in both strip and MJ treated samples.

### Functional classification of differentially expressed transcripts

**Fig. 7:**
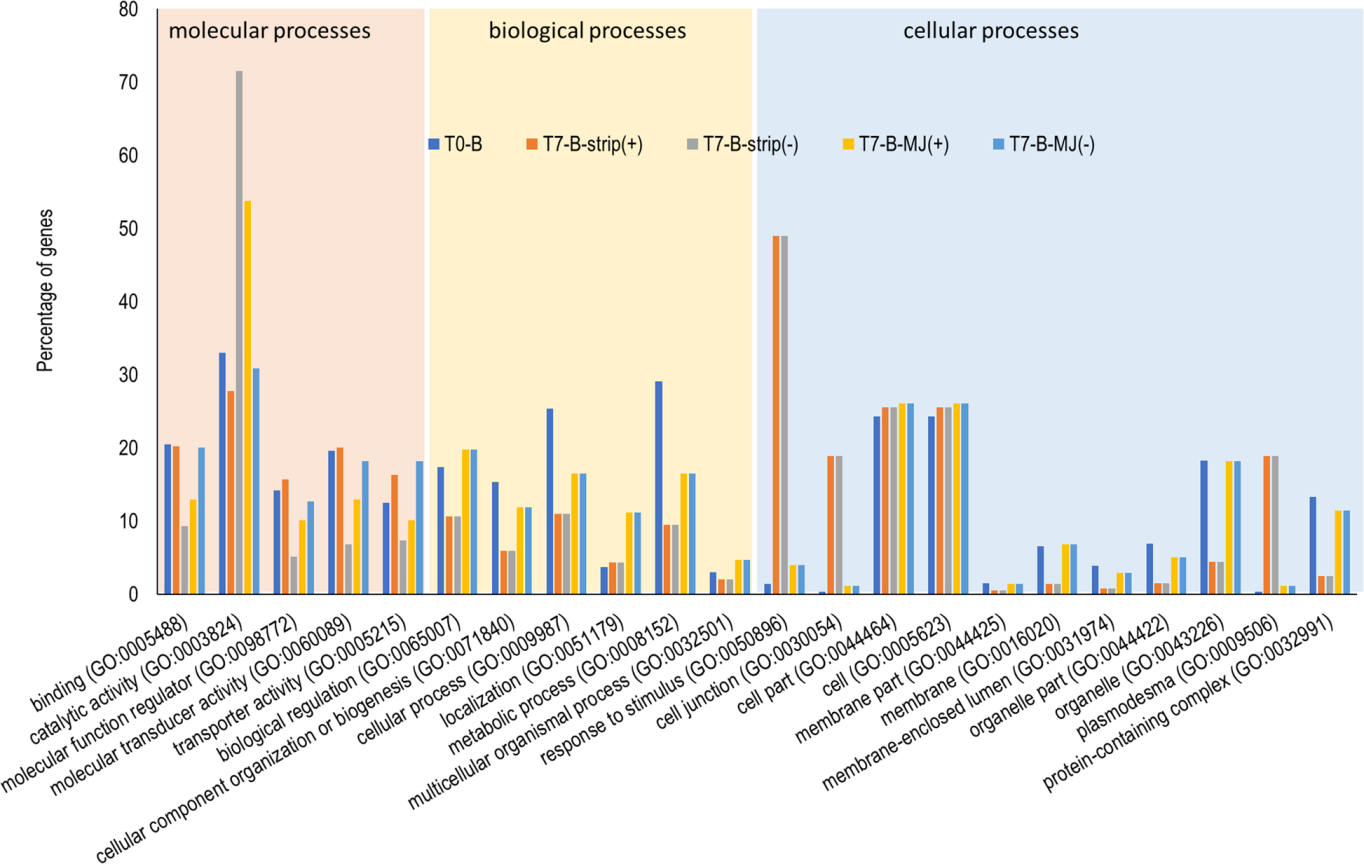
Number of transcripts in each molecular, biological and cellular categorization of up-regulated and down-regulated genes in *Pinus radiata* bark (B) at T0 and after treatment with methyl jasmonate (MJ) or bark stripping (strip) at T7. The categorization is based on gene ontology (GO) annotations of the top 100 differentially expressed transcripts in each category. GO terms with < 2% gene enrichment were excluded. (−) = down- regulated, (+) = up-regulated transcripts

To assess the overall effect of the treatments across different gene families and molecular processes, the GO terms were determined for the up-regulated and down-regulated transcripts for each condition (time × treatment × plant part). There was an overall similarity in the GO terms for genes that were up- and down-regulated in the strip and methyl jasmonate treatments. For example, in the GO-molecular processes, differentially expressed genes were associated with catalytic activity both in the needles and the bark (Fig. 7, Supplementary Fig. 1). However, the proportion of the 100 top differentially expressed genes in the catalytic activity category varied markedly. For example in the bark, a great percentage of top down-regulated genes following bark stripping were in the catalytic activity category (72%) compared with the up-regulated genes (28%).

Comparing GO terms for the top differentially expressed genes in the constitutive (needles versus bark) and induced transcriptome, indicated that some gene functions that were not strongly expressed in the constitutive state (T0) were notably up-regulated or down-regulated after treatment, and this differential expression appears to be treatment specific (Fig. 7). For example, genes related to response to stimulus (GO:0050896), plasmodesma (GO:0009506) and cell junction (GO:0030054) were strongly up-regulated at T7 in the transcriptome of the bark stripped samples but not the methyl jasmonate samples. Accordingly, transcripts of many of the other GO categories were under expressed in the transcriptome of the bark stripped samples.

## Discussion

We aimed to understand the differences in the constitutive needle and bark transcriptomes, the changes that occur following bark stripping and how they compare with those of methyl jasmonate that have been most commonly reported for conifer species [17, 24, 35, 80]. While the results are based on a partial transcriptome, comparing the needle and bark transcriptome as assessed prior to treatment (T0) showed that there were minimal qualitative differences in terms of the transcripts found in the plant parts. However, after treatment there was strong transcriptional response of the basal transcripts in both the needles and the bark, with the expression being different and with sometimes non-overlapping transcripts between plant parts, treatments and at each sampling timepoint. While the effects of methyl jasmonate have been previously reported in various pine species [17, 24], this is the first study to illustrate transcriptional responses to bark stripping. The response to bark stripping was less than that to methyl jasmonate and was localised, as no systemic response extending to the needles was detected at any time point. Differences in responsiveness to both treatments were also detected between the classes of genes, where genes related to primary metabolism responded to treatments with a greater magnititude of up-regulation or down-regulation compared to genes associated with secondary metabolism.

Among the genes that were homogeneously expressed between the bark and the needles were those related to basic life functions especially those related to primary and secondary metabolism. For example, ribulose bisphosphate carboxylase/oxygenase (RuBisCO) and a chlorophyll a/b binding protein were dominant both in the transcriptome of the needles and the bark. Similar observations were made in the needles of other *P. radiata* populations [81] and *Pinus monticola* [70], although these studies did not analyse how the transcriptomes change with treatment and the observations were limited to one plant part. Genes directly related to secondary metabolism, for example chalcone synthases, dehydrins and defensins, were among the basal genes, highlighting the importance of constitutive defences in *P. radiata*. Chalcone synthase has been identified in other conifers [82, 83] and plays crucial role in phenolic biosynthesis [74]. Defensins have also been detected in various conifers where they inhibit the growth of a broad range of pathogens, including bacteria, fungi and viruses [75, 76]. Dehydrins that represent a family of genes for drought tolerance have been detected in spruces and in other *Pinaceae* [72]. Metallothioneins that were strongly expressed both in the bark and the needles are important in protection against heavy metal toxicity [73] and have been documented mainly in *Pseudotsuga menziesii* [84, 85]. They could reflect an adaptation to leached, heavy metal enriched soils in the coastal sites of California where *P. radiata* originates [86]. However, while the above genes are expressed at high amounts equally in the bark and needles, some transcripts were up-regulated in the needles or the bark. More up-regulation was detected in the bark, which contrasted with the higher expression of transcripts in the needles than the bark reported in other *P. radiata* populations [81]. In both plant parts up-regulated genes were predominantly related to the synthesis and transfer of macro- and micro-molecules, as well as transcription factors which are the key molecular switches orchestrating the regulation of plant responses to a variety of stresses.

After treatment with methyl jasmonate and bark stripping, there was an up-regulation and down-regulation of several genes involved in both primary and secondary metabolism both in the bark and needles, consistent with other studies that have characterised responses to other stressors in conifers [24, 79]. The top genes that were up- or down-regulated in the present study overlap with those observed in similar studies with contrasting sources of stress in conifers [13, 70, 79, 80, 87], suggesting that changes in gene expression following stress are relatively conserved. Among the top expressed genes, results showed a down-regulation of hexokinases, granule-bound starch synthase and sodium-bile acid cotransporter as well as genes related with photosynthesis, suggesting reduction in sugar metabolism in the treated plants. However, cell wall invertase that mediates export of sucrose or enhanced import of hexoses at the site of damage was up-regulated in both methyl jasmonate and strip treated plants. Cell wall invertase (CWI) is an enzyme that cleaves sucrose, the major transport sugar in plants, irreversibly yielding glucose and fructose, which can be taken up by plant cells [78, 88]. An increase in CWI should ideally lead to a reduction in sucrose, which is consistent with the drastic reduction in the amounts of sucrose that has been observed following methyl jasmonate and strip treatments in *P. radiata*. The up-regulation of CWI would also suggest an increase of glucose and fructose, but this was not the case as a strong reduction in the amounts of glucose and fructose was observed in treated samples [50]. This suggests that although fructose and glucose may be potentially enhanced by an increased break down of sucrose, their utilisation for energy and carbon skeletons for other organic compounds or for tissue recovery exceeds their production, supporting the concept that defence is costly in terms of energy [89]. Gould, Reglinski [90] detected a repression of photosynthesis in *P. radiata* as a response to stress that could lead to a reduction of sugars. Sugars have also been shown to function as signalling molecules, in a manner similar to hormones [88, 91], but their down-regulation contrasts to the up-regulation of other signalling molecules. However, according to Eveland and Jackson [92] sugar signals are generated either by relative ratios to other metabolites, such as C:N, not necessarily carbohydrate concentration.

In addition to the sugar-related genes, the other primary metabolism genes that were responsive to the treatment included those genes related to fatty acid metabolism such as the medium-chain-fatty-acid-CoA ligase and UDP-rhamnose:rhamnosyltransferase that were up-regulated and those related to fatty acid hydrolysis, such as carboxylesterase, that were down-regulated. Observations on the same population showed a reduction in fatty acids following treatment, consistent with their potential use as precursors to the formation of secondary compounds [93]. Accumulating evidence has suggested lipids and lipid metabolites as important regulators of plant defence [94]. Genes related to amino acid synthesis were also among the top expressed genes. Increase in amino acid levels have been detected in plants under stress and is hypothesized to protect plant cells against dehydration [95, 96]. Amino acid accumulation has been observed to be strongly related to abscisic acid signalling [95]. Molecules related to abscisic acid signalling were also strongly up-regulated similar with pathogenicity response in the *Pinus pinaster - Fusarium circinatum* pathosystem [97]. This study contributes to the body of literature demonstrating the crucial role of phytohormones in host defense response [98].

Genes related directly to secondary metabolism were not detected among the top differentially expressed genes following treatment although they are abundant in the constitutive transcriptome of both the needles and the bark, consistent with the observations in spruce [10]. However, the relatively weak transcriptional response to treatment of individual genes related to secondary metabolism in this study contrasts with other studies [13, 17] and could possibly be due to the timing of the sampling, which was done 7 days after treatment application. In various studies, maximum expression of genes is shown to be attained within 5 days after treatment application [13, 17]. On the same population, a weak response of terpenes and phenolics was observed following similar treatments  [50], which probably suggests an inherently weak response of secondary compounds and associated genes to stress in *P. radiata*. Defence genes being strongly expressed in the constitutive but not in the induced transcriptome may suggest existence of trade-offs in induced gene expression [99], analogous to the trade-offs in constitutive versus induced chemical responses that have been detected in *P. radiata* [21]. Although alkaloids have not been well researched as important defence compounds in conifers, genes related to alkaloid biosynthesis such as RS-norcoclaurine 6-O-methyltransferase were among the top expressed genes but were down-regulated after treatment. There were also many proteins of unknown functions that were up-regulated or down-regulated at various time points, which potentially explains the many unknown chemical compounds that were quantified on the same plants.

Considerable overlap was observed between the methyl jasmonate and the strip induced transcriptome. However, results also indicate that bark stripping can induce transcripts that are not induced with methyl jasmonate and vice versa. Defence responses for bark stripping may differ from methyl jasmonate since bark stripping causes tissue and water loss at the injured sites, and damaged plants are also easily infected by pathogens through these wounds. In this case both defence and repair responses are required. Hence the dominant genes in the strip-induced transcriptome involved pathogenesis-related (PR) genes and those related to fibre synthesis. The expression of PR genes could also be related to the historical relationship between *P. radiata* and various pathogens [100]. No systemic transcript responses were observed in the needles to bark stripping. Coupled with the chemical changes that were observed in the needles following bark stripping on the same population, for example the reduction of glucose and fructose at T7 and T14 [50], this observation suggests that some chemical stress responses, possibly those involving sugars, may not involve on-site gene expression changes and may result from passive reallocation of chemistry within the plant. For other compounds like terpenes, it has been indicated that passive changes normally occur only in the constitutive environment and that stress-induced changes in terpenes are entirely of a de novo nature [101].

A key finding from this study is that the main transcriptome change associated with either treatment was clearly earlier than the main chemical changes observed on the same population [50]. The maximum differential expression of the transcripts was observed 7 days after treatment whereas most chemical change were detected 14 and 21 days after treatment, consistent with a time-lag between gene and phenotypic expression. This discrepancy may be associated with trade-offs between gene expression and other cellular resources, including the nutritional quality of the plant [99]. One GO-term that was significantly enriched after treatment was *response to stimuli* and, consistently, genes related to signalling were among the top expressed genes. For example, 1-aminocyclopropane-1-carboxylate oxidase, which is related to production of ethylene; lanC-like protein 2-like for abscissic acid and Tify domain containing protein for jasmonates were strongly responsive. Ethylene is one of the major signalling molecules in plant defences in addition to others, such as jasmonic acid, salicylic acid and abscisic acid [102]. Ethylene can act synergistically or antagonistically with jasmonic acid in the regulation of both stress and developmental responses. The connection between these two signalling pathways has been demonstrated genetically to be the transcription factor for the ethylene response [103], that was also strongly expressed. This suggests that jasmonates, abscisic acid and ethylene are involved in induced responses of *P. radiata* under different stresses. The involvement of jasmonates and ethylene in induced defence responses has been shown in other pine species [20]. In other species, abscisic acid has been shown to be involved in defence responses and has been reported to play a negative role in the regulation of the major photosynthesis gene — type 2 light-harvesting chlorophyll a/b-binding polypeptide [71] — which was reduced after treatment in this current study.

## Conclusion

There are marked quantitative differences in the needle and bark transcriptome of *Pinus radiata* both in the constitutive and induced states. The transcriptome triggered by bark stripping substantially differed from methyl jasmonate triggered responses suggesting that some molecular aspects of bark stripping may differ from other biotic and abiotic responses. Gene annotation revealed that some of the differentially expressed transcripts have putative functions in plant defence signalling, transcription regulation, biosyntheses of primary and secondary metabolites and other biological processes. The diversity of these genes reflects the complexity of stress responses. The expressed genes provide a basis for further identification of candidate genes that affect bark stripping through variation in their expression levels while the uncharacterized genes that responded to simulated herbivory and methyl jasmonate provide a rich resource for future studies. Gene expression can be used by breeders to exploit phenotype variability among individuals within or between populations. It also remains to be tested whether variations in the transcript levels, particularly the differentially expressed components in reponse to the artificial stress treatments can be linked to the susceptibility classes identified in the field [46] and whether they are heritable.

## Supplementary Information

**Additional file 1: Supplementary Figure 1.** Number of transcripts in each cellular, biological and cellular categorization of up-regulated and down-regulated genes in *Pinus radiata* needles (N) at T0 and after treatment with methyl jasmonate (MJ) or bark stripping (strip) at T7. The categorization is based on gene ontology (GO) annotations of the top 100 differentially expressed transcripts in each category. Go terms with < 2% gene enrichment were excluded. (−) = down- regulated, (+) = up-regulated transcripts.

## Data Availability

The datasets supporting the results of this article are available on reasonable request from Assoc. Prof Julianne O’Reilly-Wapstra, School of Natural Sciences, University of Tasmania, Australia. The expressed transcripts can be accessed on the ncbi website (Sequence Read Archive (SRA) submission: SUB10571957).

## References

[1] Anderson JT, Mitchell-Olds T (2011). Ecological genetics and genomics of plant defenses: evidence and approaches. Funct Ecol.

[2] Mitchell-Olds T, Willis JH, Goldstein DB (2007). Which evolutionary processes influence natural genetic variation for phenotypic traits?. Nat Rev Genet.

[3] Nantongo JS (2016). Detection of self incompatibility genotypes in Prunus africana: characterization, evolution and spatial analysis. PLoS One.

[4] Eldar A (2009). Partial penetrance facilitates developmental evolution in bacteria. Nature.

[5] Raj A (2010). Variability in gene expression underlies incomplete penetrance. Nature.

[6] Li Z (2019). Integrating gene expression data into genomic prediction. Front Genet.

[7] Guo Z (2016). Evaluation of the utility of gene expression and metabolic information for genomic prediction in maize. Theor Appl Genet.

[8] D’Agui H (2016). Phenotypic variation and differentiated gene expression of Australian plants in response to declining rainfall. R Soc Open Sci.

[9] Lamara M (2018). Association genetics of acetophenone defence against spruce budworm in mature white spruce. BMC Plant Biol.

[10] Verne S (2011). Global transcriptome analysis of constitutive resistance to the white pine weevil in spruce. Genome Biol Evol.

[11] Whitehill JG, Yuen MM, Bohlmann J. Constitutive and insect-induced transcriptomes of weevil-resistant and susceptible Sitka spruce. Plant Environ Interact. 2021; 2(3):137-147. 10.1002/pei3.10053 PMC10168040 37283859

[12] Kovalchuk A (2019). Dual RNA-seq analysis provides new insights into interactions between Norway spruce and necrotrophic pathogen Heterobasidion annosum s.l. BMC Plant Biol.

[13] Ralph SG (2006). Conifer defence against insects: microarray gene expression profiling of Sitka spruce (Picea sitchensis) induced by mechanical wounding or feeding by spruce budworms (Choristoneura occidentalis) or white pine weevils (Pissodes strobi) reveals large-scale changes of the host transcriptome. Plant Cell Environ.

[14] Behringer D (2015). Differential gene expression reveals candidate genes for drought stress response in Abies alba (Pinaceae). PLoS One.

[15] Ranade SS, Delhomme N, Garcia-Gil MR (2019). Global gene expression analysis in etiolated and de-etiolated seedlings in conifers. PLoS One.

[16] Cronn R (2017). Transcription through the eye of a needle: daily and annual cyclic gene expression variation in Douglas-fir needles. BMC Genomics.

[17] Liu J-J (2017). Profiling methyl jasmonate-responsive transcriptome for understanding induced systemic resistance in whitebark pine (Pinus albicaulis). Plant Mol Biol.

[18] Duan C (2010). Gene expression pattern in response to wounding, methyl jasmonate and ethylene in the bark of Hevea brasiliensis. Tree Physiol.

[19] Hudgins J, Christiansen E, Franceschi VR (2003). Methyl jasmonate induces changes mimicking anatomical defenses in diverse members of the Pinaceae. Tree Physiol.

[20] Hudgins JW, Franceschi VR (2004). Methyl jasmonate-induced ethylene production is responsible for conifer phloem defense responses and reprogramming of stem cambial zone for traumatic resin duct formation. Plant Physiol.

[21] Moreira X (2014). Trade-offs between constitutive and induced defences drive geographical and climatic clines in pine chemical defences. Ecol Lett.

[22] Kant MR (2015). Mechanisms and ecological consequences of plant defence induction and suppression in herbivore communities. Ann Bot.

[23] Miller B (2005). Insect-induced conifer defense. White pine weevil and methyl jasmonate induce traumatic resinosis, de novo formed volatile emissions, and accumulation of terpenoid synthase and putative octadecanoid pathway transcripts in Sitka spruce. Plant Physiol.

[24] Kānberga-Siliņa K (2017). Transcriptomic response to methyl jasmonate treatment of scots pine (Pinus sylvestris) seedlings. Environ Exp Biol.

[25] Litvak ME, Monson RK (1998). Patterns of induced and constitutive monoterpene production in conifer needles in relation to insect herbivory. Oecologia.

[26] Kolosova N (2010). Transcriptome analysis of conifer defense against bark beetle-associated blue-stain fungi and white pine weevil. Botany.

[27] Zulak KG, Bohlmann J (2010). Terpenoid biosynthesis and specialized vascular cells of conifer defense. J Integr Plant Biol.

[28] Du M, Ding G, Cai Q (2018). The transcriptomic responses of Pinus massoniana to drought stress. Forests.

[29] Byun-McKay A (2006). Wound-induced terpene synthase gene expression in Sitka spruce that exhibit resistance or susceptibility to attack by the white pine weevil. Plant Physiol.

[30] Keeling CI, Bohlmann J (2006). Genes, enzymes and chemicals of terpenoid diversity in the constitutive and induced defence of conifers against insects and pathogens. New Phytol.

[31] Reymond P (2004). A conserved transcript pattern in response to a specialist and a generalist herbivore. Plant Cell.

[32] Kovalchuk A (2015). Activation of defence pathways in Scots pine bark after feeding by pine weevil (Hylobius abietis). BMC Genomics.

[33] Martin D (2002). Methyl jasmonate induces traumatic resin ducts, terpenoid resin biosynthesis, and terpenoid accumulation in developing xylem of Norway spruce stems. Plant Physiol.

[34] Korth KL (2003). Profiling the response of plants to herbivorous insects. Genome Biol.

[35] Men L, Yan S, Liu G (2013). De novo characterization of Larix gmelinii (Rupr.) Rupr. transcriptome and analysis of its gene expression induced by jasmonates. BMC Genomics.

[36] Ohse B (2017). Salivary cues: simulated roe deer browsing induces systemic changes in phytohormones and defence chemistry in wild-grown maple and beech saplings. Funct Ecol.

[37] Agrawal AA (2000). Specificity of induced resistance in wild radish: causes and consequences for two specialist and two generalist caterpillars. Oikos.

[38] Cukor J (2019). Effects of bark stripping on timber production and structure of Norway spruce forests in relation to climatic factors. Forests.

[39] Nagaike T. Effects of heavy, repeated bark stripping by Cervus nippon on survival of Abies veitchii in a subalpine coniferous forest in central Japan. J For Res. 2019; 31:1139–1145.

[40] ABARES (2018). Australia’s state of the forests report.

[41] Eldridge KG (1979). Seed collections of *Pinus radiata* and *P. muricata* in California. Forest genetic resources information.

[42] Nantongo JS (2020). Quantitative genetic variation in bark stripping of *Pinus radiata*. Forests.

[43] Page DE (2013). Seasonal dynamics in understorey abundance and carbohydrate concentration in relation to browsing and bark stripping of Tasmanian *Pinus radiata* plantations. For Ecol Manag.

[44] Miller A, O’Reilly-Wapstra J, Potts B (2014). Genetic variation in bark stripping among *Pinus radiata*.

[45] Smith AH (2020). Ease of access to an alternative food source enables wallabies to strip bark in Tasmanian *Pinus radiata* plantations. Forests.

[46] Nantongo JS, et al. Chemical traits that predict susceptibility of *Pinus radiata* to marsupial bark stripping. J Chem Ecol. 2021. 10.1007/s10886-021-01307-5. 10.1007/s10886-021-01307-5 34611747

[47] Nantongo JS (2021). Additive genetic variation in *Pinus radiata* bark chemistry and the chemical traits associated with variation in mammalian bark stripping. Heredity.

[48] Lundborg L (2019). Effects of methyl jasmonate on the concentration of volatile terpenes in tissues of Maritime pine and Monterey pine and its relation to pine weevil feeding. Trees.

[49] Moreira X, Zas R, Sampedro L (2012). Differential allocation of constitutive and induced chemical defenses in pine tree juveniles: a test of the optimal defense theory. PLoS One.

[50] Nantongo JS, et al. Variation in constitutive and induced chemistry in the needles, bark and roots of *Pinus radiata*. Trees. 2021; doi.org/10.1007/s00468-021-02209-5

[51] Reglinski T (2017). Biochemical responses associated with induced resistance to Colletotrichum acutatum in *Pinus radiata* seedlings treated with methyl jasmonate and Trichoderma spp. For Pathol.

[52] Nantongo JS (2021). Developing near infrared spectroscopy models for predicting chemistry and responses to stress in *Pinus radiata* (D. Don). J Near Infrared Spectrosc.

[53] Alvarez C (2011). Variation in gene expression profile with aging of *Pinus radiata* D. Don. BMC Proc.

[54] Telfer E (2018). Approaches to variant discovery for conifer transcriptome sequencing. PLoS One.

[55] Li X, Yang X, Wu HX (2013). Transcriptome profiling of radiata pine branches reveals new insights into reaction wood formation with implications in plant gravitropism. BMC Genomics.

[56] Carrasco A (2017). Expression profiling in *Pinus radiata* infected with Fusarium circinatum. Tree Genet Genomes.

[57] Moreira X (2013). Inducibility of chemical defences by two chewing insect herbivores in pine trees is specific to targeted plant tissue, particular herbivore and defensive trait. Phytochemistry.

[58] Andrews S (2018). FastQC: a quality control tool for high throughput sequence data. Babraham bioinformatics.

[59] Bolger AM, Lohse M, Usadel B (2014). Trimmomatic: a flexible trimmer for Illumina sequence data. Bioinformatics.

[60] Ewing B, Green P (1998). Base-calling of automated sequencer traces using phred. II. Error probabilities. Genome Res.

[61] Grabherr MG (2011). Full-length transcriptome assembly from RNA-Seq data without a reference genome. Nat Biotechnol.

[62] Patro R (2017). Salmon provides fast and bias-aware quantification of transcript expression. Nat Methods.

[63] R Core Team (2018). R: a language and environment for statistical computing.

[64] Robinson MD, McCarthy DJ, Smyth GK (2010). edgeR: a Bioconductor package for differential expression analysis of digital gene expression data. Bioinformatics.

[65] Soneson C, Delorenzi M (2013). A comparison of methods for differential expression analysis of RNA-seq data. BMC Bioinformatics.

[66] Aubert J (2004). Determination of the differentially expressed genes in microarray experiments using local FDR. BMC bioinformatics.

[67] Lê S, Josse J, Husson F (2008). FactoMineR: an R package for multivariate analysis. J Stat Softw.

[68] Bairoch A, Apweiler R (2000). The SWISS-PROT protein sequence database and its supplement TrEMBL in 2000. Nucleic Acids Res.

[69] Mi H (2019). PANTHER version 14: more genomes, a new PANTHER GO-slim and improvements in enrichment analysis tools. Nucleic Acids Res.

[70] Liu J-J, Sturrock RN, Benton R (2013). Transcriptome analysis of Pinus monticola primary needles by RNA-seq provides novel insight into host resistance to Cronartium ribicola. BMC Genomics.

[71] Liu R (2013). Light-harvesting chlorophyll a/b-binding proteins, positively involved in abscisic acid signalling, require a transcription repressor, WRKY40, to balance their function. J Exp Bot.

[72] Stival Sena J (2018). Expansion of the dehydrin gene family in the Pinaceae is associated with considerable structural diversity and drought-responsive expression. Tree Physiol.

[73] Nevrtalova E (2014). Expression response of duplicated metallothionein 3 gene to copper stress in Silene vulgaris ecotypes. Protoplasma.

[74] Dixon RA, Paiva NL (1995). Stress-induced phenylpropanoid metabolism. Plant Cell.

[75] Picart P (2012). Identification of defensin-encoding genes of Picea glauca: characterization of PgD5, a conserved spruce defensin with strong antifungal activity. BMC Plant Biol.

[76] Ermakova EA (2016). Structure of Scots pine defensin 1 by spectroscopic methods and computational modeling. Int J Biol Macromol.

[77] The UniProt Consortium (2018). UniProt: a worldwide hub of protein knowledge. Nucleic Acids Res.

[78] Proels RK, Hückelhoven R (2014). Cell-wall invertases, key enzymes in the modulation of plant metabolism during defence responses. Mol Plant Pathol.

[79] Liu Q (2017). Transcriptomic profiling reveals differentially expressed genes associated with pine wood nematode resistance in masson pine (Pinus massoniana Lamb.). Sci Rep.

[80] Celedon JM (2017). Cell-type- and tissue-specific transcriptomes of the white spruce (Picea glauca) bark unmask fine-scale spatial patterns of constitutive and induced conifer defense. Plant J.

[81] Alvarez C (2016). Changes in gene expression in needles and stems of *Pinus radiata* rootstock plants of different ontogenic age. Am J Plant Sci.

[82] Richard S (2000). Induction of chalcone synthase expression in white spruce by wounding and jasmonate. Plant Cell Physiol.

[83] Baker SM, White EE (1996). A chalcone synthase/stilbene synthase DNA probe for conifers. Theor Appl Genet.

[84] Chatthai M (2004). Functional analysis of a Douglas-fir metallothionein-like gene promoter: transient assays in zygotic and somatic embryos and stable transformation in transgenic tobacco. Planta.

[85] Chatthai M (1997). The isolation of a novel metallothionein-related cDNA expressed in somatic and zygotic embryos of Douglas-fir: regulation by ABA, osmoticum, and metal ions. Plant Mol Biol.

[86] Keator G (2002). Introduction to trees of the San Francisco bay region. California natural history guides.

[87] Fox H (2017). Transcriptome analysis of Pinus halepensis under drought stress and during recovery. Tree Physiol.

[88] Tauzin AS, Giardina T (2014). Sucrose and invertases, a part of the plant defense response to the biotic stresses. Front Plant Sci.

[89] Gershenzon J (1994). Metabolic costs of terpenoid accumulation in higher plants. J Chem Ecol.

[90] Gould N (2008). Physiological trade-offs associated with methyl jasmonate-induced resistance in *Pinus radiata*. Can J For Res.

[91] Trouvelot S (2014). Carbohydrates in plant immunity and plant protection: roles and potential application as foliar sprays. Front Plant Sci.

[92] Eveland AL, Jackson DP (2012). Sugars, signalling, and plant development. J Exp Bot.

[93] Kachroo A, Kachroo P (2009). Fatty acid–derived signals in plant defense. Annu Rev Phytopathol.

[94] Shah J (2005). Lipids, lipases, and lipid-modifying enzymes in plant disease resistance. Annu Rev Phytopathol.

[95] Al-Asbahi AA, Al-Maqtari MA, Naji KM (2012). ABA biosynthesis defective mutants reduce some free amino acids accumulation under drought stress in tomato leaves in comparison with Arabidopsis plants tissues. J Stress Physiol Biochem.

[96] Joshi V, Jander G (2009). Arabidopsis methionine γ-lyase is regulated according to isoleucine biosynthesis needs but plays a subordinate role to threonine deaminase. Plant Physiol.

[97] Hernandez-Escribano L (2020). The transcriptome of Pinus pinaster under Fusarium circinatum challenge. BMC Genomics.

[98] Pieterse CMJ (2012). Hormonal modulation of plant immunity. Annu Rev Cell Dev Biol.

[99] Kim J (2020). Trade-offs between gene expression, growth and phenotypic diversity in microbial populations. Curr Opin Biotechnol.

[100] Offord HR (1964). Diseases of Monterey pine in native stands of California and in plantations of western North America.

[101] Wu C (2017). ^13^C labelling study of constitutive and stress-induced terpenoide missions from Norway spruce and Scots pine. Biogeosciences.

[102] Yang J (2019). The crosstalks between jasmonic acid and other plant hormone signaling highlight the involvement of jasmonic acid as a core component in plant response to biotic and abiotic stresses. Front Plant Sci.

[103] Lorenzo O (2003). Ethylene response Factor1 integrates signals from ethylene and jasmonate pathways in plant defense. Plant Cell.

