# Supplementary material for: Analysis of the transcriptome of the needles and bark of Pinus radiata induced by bark stripping and methyl jasmonate
Source: BMC Genomics. 2022 Jan 13;23:52. doi: 10.1186/s12864-021-08231-8 (PMC8759178; doi:10.1186/s12864-021-08231-8)
Supplement: Supplementary file 1 — Additional file 1: Supplementary Figure 1. Number of transcripts in each cellular, biological and cellular categorization of up-regulated and down-regulated genes in Pinus radiata needles (N) at T0 and after treatment with methyl jasmonate (MJ) or bark stripping (strip) at T7. The categorization is based on gene ontology (GO) annotations of the top 100 differentially expressed transcripts in each category. Go terms with < 2% gene enrichment were excluded. (−) = down- regulated, (+) = up-regulated transcripts. [file 12864_2021_8231_MOESM1_ESM.docx]

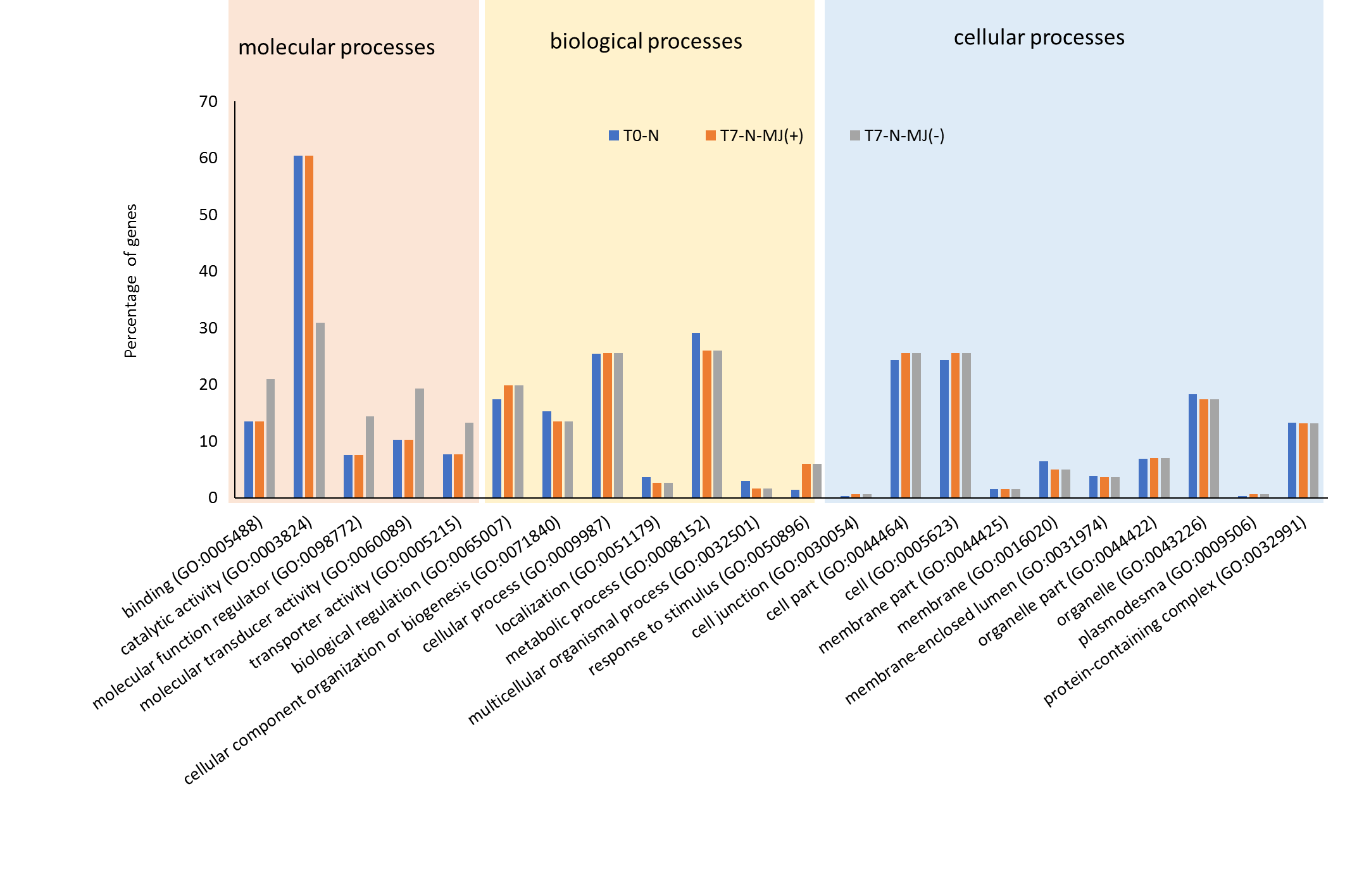


**Supplementary Figure 1**: Number of transcripts in each cellular, biological and cellular categorization of up-regulated and down-regulated genes in *Pinus radiata* needles (N) at T0 and after treatment with methyl jasmonate (MJ) or bark stripping (strip) at T7. The categorization is based on gene ontology (GO) annotations of the top 100 differentially expressed transcripts in each category. Go terms with <2% gene enrichment were excluded. (-) = down- regulated, (+) = up-regulated transcripts
